# Genome‐wide analyses suggest parallel selection for universal traits may eclipse local environmental selection in a highly mobile carnivore

**DOI:** 10.1002/ece3.1695

**Published:** 2015-09-22

**Authors:** Astrid Vik Stronen, Bogumiła Jędrzejewska, Cino Pertoldi, Ditte Demontis, Ettore Randi, Magdalena Niedziałkowska, Tomasz Borowik, Vadim E. Sidorovich, Josip Kusak, Ilpo Kojola, Alexandros A. Karamanlidis, Janis Ozolins, Vitalii Dumenko, Sylwia D. Czarnomska

**Affiliations:** ^1^ Section of Biology and Environmental ScienceDepartment of Chemistry and BioscienceAalborg UniversityFredrik Bajers Vej 7HDK‐9220Aalborg ØstDenmark; ^2^ Mammal Research InstitutePolish Academy of Sciencesul. Waszkiewicza 1PL 17‐230BialowiezaPoland; ^3^ Aalborg ZooMølleparkvej 63DK‐9000AalborgDenmark; ^4^ Department of Human GeneticsUniversity of AarhusWilhelm Meyers AlléDK‐8000AarhusDenmark; ^5^ Laboratorio di GeneticaISPRAvia Cà Fornacetta 9I‐40064Ozzano Emilia (BO)Italy; ^6^ Institute of ZoologyScientific and Practical Centre for Biological ResourcesNational Academy of Science of BelarusAkademicheskaya Str 27220072MinskBelarus; ^7^ Department of BiologyFaculty of Veterinary MedicineUniversity of ZagrebZagrebCroatia; ^8^ Natural Resources Institute FinlandBox 16FI‐96500RovaniemiFinland; ^9^ ARCTUROSCivil Society for the Protection and Management of Wildlife and the Natural EnvironmentGR‐53075AetosGreece; ^10^ Department of Ecology and Natural Resources ManagementNorwegian University of Life SciencesNO‐1432ÅsNorway; ^11^ Latvian State Forest Research Institute “Silava”Rīgas 111LV‐2169SalaspilsLatvia; ^12^ Biosphere Reserve Askania NovaFrunze Str. 13Askania‐NovaChaplynka DistrictKherson Region75230Ukraine

**Keywords:** CanineHD BeadChip microarray, *Canis lupus*, environmental selection, genome‐wide association study, single nucleotide polymorphism, wolf

## Abstract

Ecological and environmental heterogeneity can produce genetic differentiation in highly mobile species. Accordingly, local adaptation may be expected across comparatively short distances in the presence of marked environmental gradients. Within the European continent, wolves (*Canis lupus*) exhibit distinct north–south population differentiation. We investigated more than 67‐K single nucleotide polymorphism (SNP) loci for signatures of local adaptation in 59 unrelated wolves from four previously identified population clusters (northcentral Europe *n* = 32, Carpathian Mountains *n* = 7, Dinaric‐Balkan *n* = 9, Ukrainian Steppe *n* = 11). Our analyses combined identification of outlier loci with findings from genome‐wide association study of individual genomic profiles and 12 environmental variables. We identified 353 candidate SNP loci. We examined the SNP position and neighboring megabase (1 Mb, one million bases) regions in the dog (*C. lupus familiaris*) genome for genes potentially under selection, including homologue genes in other vertebrates. These regions included functional genes for, for example, temperature regulation that may indicate local adaptation and genes controlling for functions universally important for wolves, including olfaction, hearing, vision, and cognitive functions. We also observed strong outliers not associated with any of the investigated variables, which could suggest selective pressures associated with other unmeasured environmental variables and/or demographic factors. These patterns are further supported by the examination of spatial distributions of the SNPs associated with universally important traits, which typically show marked differences in allele frequencies among population clusters. Accordingly, parallel selection for features important to all wolves may eclipse local environmental selection and implies long‐term separation among population clusters.

## Introduction

Local adaptation may be predicted in areas with limited influx of novel genes, which can interrupt selection for local environmental conditions, or in regions of high gene flow countered by strong selective pressures (Slatkin 1987 and references therein). An alternate explanation is selective dispersal with genotypes preadapted to the local environment – or natal habitat‐biased dispersal (Davis and Stamps 2004; Nosil et al. 2005; Edelaar et al. 2008) – a process that may help explain local adaptation in highly mobile organisms with broad geographic distributions. Ecological and environmental differentiation can cause population genetic structure in highly mobile species (Davis and Stamps 2004; Nosil et al. 2005), whereby dispersers select habitat conditions for which they have natal experience and are better able to survive and reproduce. Accordingly, genetic divergence may be expected across comparatively short geographic distances in the presence of marked environmental gradients if genetic drift is not overwhelming the selective forces. Long‐term responses to selection in a finite population are also influenced by factors dependent on the effective population size and population structure (De Souza et al. 2000; Pertoldi et al. 2007).

Although long‐distance gene flow occurs sufficiently often to produce genetic homogeneity over a wide geographic range (Slatkin 1985), new findings imply that ecological and environmental variation can result in genetic differentiation across taxa including wide‐ranging terrestrial and marine species. Examples include fish such as herring (*Clupea harengus*, André et al. 2011), hake (*Merluccius merluccius*, Milano et al. 2014), and Baltic Sea stickleback (*Gasterosteus aculeatus*, DeFaveri et al. 2013); sea turtles (reviewed in Bowen and Karl 2007); and mammals including orca (*Orcinus orca*, Hoelzel et al. 2007), cougar (*Puma concolor*, McRae et al. 2005), lynx (*Lynx canadensis*, Rueness et al. 2003), and coyote (*Canis latrans*, Sacks et al. 2004, 2005). The understanding of local adaptation therefore has implications across the taxonomic range including wild species and domestic animals (e.g., Pariset et al. 2009).

Whereas carnivores are highly mobile, they can exhibit marked population genetic structure that may have important evolutionary implications. Preference for natal habitats is proposed to explain population structure in one of the most mobile and widely distributed species of large carnivores, the gray wolf (*Canis lupus*, Carmichael et al. 2001; Weckworth et al. 2005, 2010, 2011; Pilot et al. 2006, 2012; Musiani et al. 2007; Muñoz‐Fuentes et al. 2009; Stronen et al. 2014). European wolves have been affected by human‐induced landscape changes that resulted in small and often isolated populations (Linnell et al. 2008) in part due to overharvesting (Randi 2011). Populations such as those of the Italian and Iberian peninsulas have been subject to a substantial amount of genetic drift due to low effective population size and demographic stochasticity (Lucchini et al. 2004; Fabbri et al. 2007; Stronen et al. 2013; Pilot et al. 2014a).

The genetic divergence between populations of wide‐ranging species has been influenced by biogeographic processes such as glaciations, and recolonization from glacial refugia may help explain differentiation between neighboring populations of wide‐ranging carnivores (e.g., Manel et al. 2004 and references therein). Wolves appear to have been common in the Eurasian Late Pleistocene faunal complex and may have been distributed throughout Europe during this time (Kahlke 1999). The occurrence of cold‐adapted prey species such as reindeer (*Rangifer tarandus*) and mammoth (*Mammuthus primigenius*) (Kahlke 1999; Sommer and Nadachowski 2006) in southern and central Europe during the last glacial maximum suggests that a wolf ecotype adapted to arctic conditions might have been widely distributed. Wolves may have been present in central Europe during the Pleni‐Glacial epoch (circa 75–15,000 BC) with dynamic range changes during the Holocene for different ecotypes adapted to conditions such as arctic tundra, forest, and humid climates (Sommer and Benecke 2005). The spatio‐temporal extent of selection in wolves may be highly complex, and the relative influence of local environmental selection since the last glacial maximum versus independent selection in previously separated populations is not well understood. For simplicity, we henceforth refer to “ancient” selection as that having occurred prior to the last glacial maximum and “recent” as having taken place afterward.

The European continent encompasses important environmental variation. The diverse geography with (partially) east–west‐oriented mountain chains (Alps, Carpathians) and the Mediterranean and Baltic Seas might exert more complex spatial influence on population structure and gene flow than that observed in North America with well‐separated coastal and continental climates (e.g., Geffen et al. 2004). European wolves showed clear population genetic structure when evaluated over 67,000 (henceforth 67 K) single nucleotide polymorphism (SNP) markers (Stronen et al. 2013), but it remains unclear whether adaptation to various environmental conditions might help explain the observed population clusters. Although some level of genetic structure seems to have been established prior to the last glacial maximum (Pilot et al. 2010), wolves likely had a continuous range through the Holocene with population fragmentation and habitat loss primarily occurring in the past few centuries (Pilot et al. 2014a). Whereas genetic drift has affected European wolves over the past hundred years, this process seems to have been less pronounced in east–central Europe where populations have remained relatively well connected (Stronen et al. 2013; Pilot et al. 2014a). Genetic drift, population demographic history and other neutral processes could be major influences on allele frequencies and distributions where selection is weak (Coop et al. 2009). We nonetheless expect genetic drift to have an overall influence across the entire genome whereas selection is predicted to act only on certain genes. Additionally, we expect the correlation between neutral molecular diversity and non‐neutral genetic variation to be weak in stable populations, and to decrease further when populations expand or decline in size (Pertoldi et al. 2007). Our study aimed to determine whether population structure associated with functional genetic variation in European wolves 1) is consistent with previously observed (and assumed predominantly “neutral”) genetic structure and 2) appears better explained by ancient selection for common traits occurring in parallel in separate populations, or by recent selection based on local environmental conditions.

## Materials and Methods

### Samples DNA extraction and genotyping

**Figure 1:**
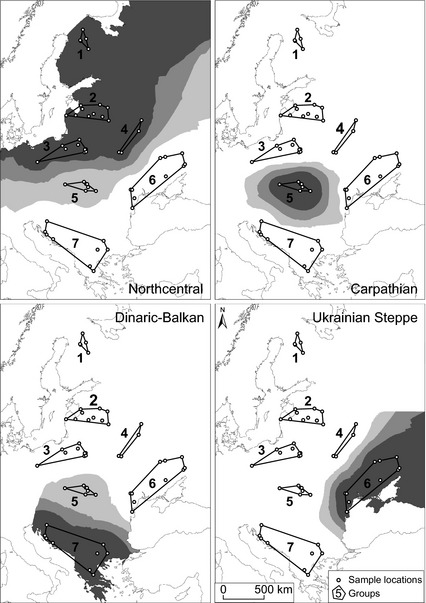
Study area and locations for 59 wolves used in analyses of single nucleotide polymorphisms (SNPs). Spatial interpolation for four SNPs with genotypes specific for different population clusters is shown as examples. The large northcentral European cluster was divided into groups 1–4 for investigation of possible regional patterns (genotype 223AA), group 5 is the Carpathian Mountains (342GA), group 6 is the Ukrainian Steppe (236AG), and group 7 is Dinaric‐Balkan (214AA). SNP allele frequencies among samples in each cluster were classified as <25% (white), 25–49% (light gray), 50–75% (medium gray), and >75% (dark gray). SNP identifications are provided in Table S2.

We examined wolf profiles from 10 countries across Europe, genotyped with the CanineHD BeadChip microarray with 170,000 SNP loci from Illumina (Illumina, Inc., San Diego, CA) as described in Stronen et al. (2013). The earlier study included Italian wolves, but owing to their highly divergent status (Stronen et al. 2013; Pilot et al. 2014a) and the possibility that strong genetic drift between Italian and other European wolves might confound signals of selection, we excluded all Italian individuals from the analyses. Moreover, we removed outlier profiles from other countries including putative wolf–dog hybrids, which resulted in a sample of *n* = 113 wolves. Subsequently, we used PLINK (Purcell et al. 2007) to identify pairs of wolves with an identity‐by‐descent (IBD, or PI_HAT) score of ≥0.1 and removed one individual per pair (some wolves had values above the threshold for multiple pairwise comparisons) to limit the potentially confounding effect of cryptic relatedness (see, e.g., Smith et al. 2010) on possible signals of selection. The screening resulted in a sample of *n* = 59 European wolves from four population clusters (northcentral Europe *n* = 32, Carpathian Mountains *n* = 7, Dinaric‐Balkan *n* = 9, Ukrainian Steppe *n* = 11, Fig. 1) previously identified by Stronen et al. (2013).

### Statistical analyses of genetic structure

We performed analyses in two stages. We first combined genome‐wide association study (GWAS, e.g., Smith et al. 2010) of genotype–environment associations in PLINK with a complimentary approach using BayeScan (Foll and Gaggiotti 2008) for detecting outlier loci without considering environmental data. We performed GWAS with 99,551 SNPs quality‐controlled and filtered for minor allele frequency and genotyping call rate (PLINK settings: maf 0.01, geno 0.02) as described in Stronen et al. (2013). For the GWAS, we included all data to retain as much information as possible for individuals and SNP loci associated with environmental factors. Subsequently, we used a 67‐K version of the data pruned for linkage disequilibrium as described in Stronen et al. (2013) to perform the BayeScan analyses carried out per population (cluster). The resulting candidate SNPs were evaluated with the spatial analysis method (SAM) implemented in the program MatSAM v2 Beta (Joost et al. 2007, 2008) because analysis involving thousands of loci was not practically feasible in MatSAM v2 Beta. However, the SAM approach is developed for analyses of genotype–environment associations in wild or domestic species (see, e.g., Pariset et al. 2009) and therefore well‐suited to the purpose of our study.

We performed GWAS in PLINK using the linear regression option, whereby each individual was assessed based on 12 environmental variables (Table 1). Environmental variables were tested for deviations from normal distribution in PAST (Hammer et al. 2001), and we log‐transformed values for which the probability plot correlation coefficient (PPCC) was <0.8. As a result, PPCC for all variables except two (log altitude = 0.83, log biome = 0.86) was >0.93. We examined correlation among environmental variables in PAST using the test option Kendall's tau for nonparametric data, which is a recommended option for data sets with many tied ranks (Legendre and Legendre 1998). We adjusted for multiple testing using the Bonferroni correction and categorized relationships between pairs of variables as highly (>0.6), moderately (0.3–0.6), or not correlated (<0.3). A priori exclusion of correlated variables (e.g., July, January, and annual temperature) might miss important information, and we chose to retain all variables and report their extent of correlation (Table S1). We evaluated the inclusion of 2–15 covariates obtained from multidimensional scaling of the data in PLINK to account for population stratification (Freedman et al. 2004 and references therein; Stronen et al. 2013) and performed GWAS with six covariates, the lowest number of covariates for which the genome‐inflation factor was <1.05 for all variables. GWAS tests were performed for the minor allele for each locus, and we implemented Bonferroni corrections for multiple testing (*P* < 0.05). We included all environmental variables for the final analysis in MatSAM, as this approach is based on logistic regression and does not require normal distributions.

**Table 1:** Environmental variables for genome‐wide association study of European wolves (*n* = 59) with 67‐K single nucleotide polymorphism (SNP) loci

Variable	Label	Unit	Data source
Longitude	long	Decimal degrees	Sample coordinates
Latitude	lat	Decimal degrees	Sample coordinates
Human population density	popd	Number of people/km^2^	1)
Mean annual temperature	annt	Degrees Celsius	2)
Mean January temperature	jant	Degrees Celsius	2)
Mean July temperature	jult	Degrees Celsius	2)
Annual precipitation	pred	mm	2)
Road density	road	km road/100 km^2^	3)
Altitude	alt	Meters above sea level	4)
Snow cover depth	snow	cm	5)
Ecosystem code	ecoc	Number (ordinal)	6)
Biome code	bioc	Number (ordinal)	6)

1) http://epp.eurostat.ec.europa.eu/portal/page/portal/eurostat/home/, March 2012.

2) Hijmans, R.J., S.E. Cameron, J.L. Parra, P.G. Jones and A. Jarvis (2005). Very high resolution interpolated climate surfaces for global land areas. International Journal of Climatology 25: 1965–1978. (WorldClim project data).

3) ESRI Data & Maps (2008). Redlands, CA: Environmental Systems Research Institute [CD‐ROM].

4) U.S. Geological Survey (2004), EROS Data Center Distributed Active Archive Center (EDC DAAC), Global Digital Elevation Model (GTOPO30), Redlands, California, USA. (GTOPO30 database).

5) Afonin, A.N., S.L. Greene, N.I. Dzyubenko, A.N. Frolov (2008) Interactive Agricultural Ecological Atlas of Russia and Neighboring Countries. Economic Plants and their Diseases, Pests and Weeds. Available at: http://www.agroatlas.ru.

6) Olson, D. M., E. Dinerstein (2002). The Global 200: Priority ecoregions for global conservation. (PDF file) Annals of the Missouri Botanical Garden 89:125–126. Available at: http://www.worldwildlife.org/science/data/terreco.cfm. (WWF database).

Subsequently, we performed simulations in BayeScan (Foll and Gaggiotti 2008) for the 67‐K SNPs to identify outlier loci. We tested various levels of prior (10, 100, and 1000) as the chosen value represents a trade‐off between false positives and the ability to detect possible outliers (Foll 2012). Because the loci identified as outliers were highly consistent among runs, we retained the results for prior of 10 and report loci for which the log10(PO) values were >0.5 as recommended in the program guidelines (Foll 2012). We ran analyses including all four population clusters (labeled 4P) and then performed comparisons between each pair of clusters (northcentral Europe = N, Carpathian Mountains = C, Dinaric‐Balkan = B, Ukrainian Steppe = U). Positive values for the parameter alpha (alpha > 0) indicate divergent selection, whereas negative values (alpha < 0) suggest balancing selection.

For the second stage of analysis, we evaluated GWAS and BayeScan candidate loci with MatSAM. The program performs tests of logistic regression for each SNP genotype (for which there are normally three: AA, AB, BB) and the environmental variable in question. The program implements two separate tests, a likelihood ratio (henceforth G) test and a Wald‐Beta test (Joost et al. 2007), to determine whether a particular genotype is associated with a given environmental variable. The program reports both test results, as well as a cumulative test. The cumulative test is significant when both Wald and G‐tests reject the null hypothesis that the model with the observed variable does not explain the observed genotype distribution better than a model with a constant only (Joost et al. 2007). The program implements the Bonferroni correction for multiple tests, and we chose a *P*‐level of 0.05. Values for two categorical variables, ecozone and biome, were entered as numbers using the “independent” design (Joost and Kalbermatten 2010).

We evaluated spatial patterns throughout the study area by plotting allele frequency distributions for all candidate loci. The large northcentral cluster was divided into four groups based on geographic proximity of sample locations to evaluate the possibility of local patterns. Interpolation maps for results that displayed geographic patterns were prepared with ArcGIS 10.2 (ESRI 2013). Samples were interpolated into continuous surfaces with the inverse distance weighted (IDW) method. The interpolation was conducted for four SNPs with genotypes specific for different population clusters. Genotype frequencies were stored in binary format (1 – present, 0 – not present), and the mean value was calculated for each cluster. For the northcentral cluster, we used the four above‐mentioned groups to assess the possible presence of local patterns. Inverse distance weighted interpolation was performed with default parameters. Single nucleotide polymorphism allele probability was classified into four groups (low – less than 25%, moderate – 25–50%, high – 50–75%, and very high – more than 75%).

Subsequently, we used the 67‐K SNPs in Genepop (Rousset 2008) to calculate *F*_ST_ values for each locus. We then employed HierFstat (Goudet 2005) to obtain pairwise *F*_ST_ values with 95% confidence intervals between population clusters for the 353 candidate loci identified in GWAS and BayeScan. We examined these population clusters by principal component analyses (PCA) with the *adegenet* package (Jombart 2008) in R 2.14.2 (R Development Core Team 2012).

## Results

We detected 178 outlier SNPs in BayeScan and 175 SNPs with putative association with environmental variables by GWAS. There was no overlap between the loci reported by each method. One hundred and seventy‐five of 178 SNPs (98%) identified by BayeScan had *F*_ST_ values ≥ 0.15 (as estimated by Genepop across the 59 wolves and 353 loci), which may be considered as a high (Balloux and Lugon‐Moulin 2002), whereas for GWAS the number of SNPs with *F*_ST_ values ≥ 0.15 was 21 of 175 (12%). Mean *F*_ST_ value for BayeScan loci was 0.305 (range 0.118–0.571), and for GWAS, it was 0.085 (range 0.000–0.432). All BayeScan results had a positive alpha value, suggesting directional rather than balancing selection. Over 66% of the BayeScan loci had a high loading (here defined as ≥ [0.01]) on one or two of the three PC axes in a PCA of the European wolf population with 67‐K loci (Stronen et al. 2013 Fig. 2B and C) and thus made an obvious contribution to population structure. GWAS loci showed no such pattern.

**Figure 2:**
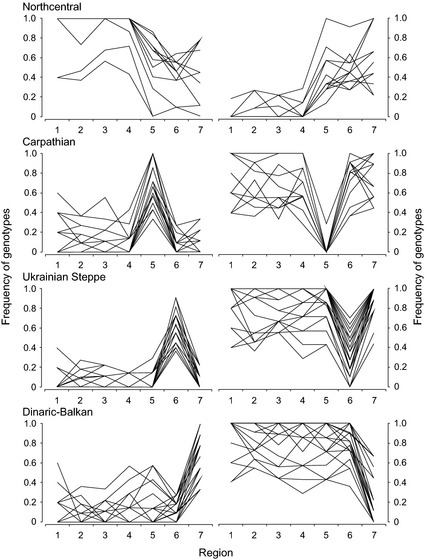
Spatial distributions of European wolf single nucleotide polymorphism (SNP) loci/genotypes typical for single population clusters. The graphs show frequencies of loci/genotypes differentiating among wolves in northcentral (groups 1–4), Carpathian (5), Ukrainian Steppe (6), and Dinaric‐Balkan clusters (group 7). Numbers on *x*‐axis are wolf groups 1–7 (see Fig. 1). Left panels: loci/genotypes with high frequencies in a given cluster. Right panels: loci/genotypes with low frequencies in a given cluster. SNP loci and genotypes are listed in Table S4.

Of the 353 SNPs, genotypes in 117 (46 from GWAS and 71 from BayeScan) were identified as associated with environmental variables by SAM. All cases in which genotypes were significantly associated with the variable “biome code” (bioc) were identified by the Wald test. No other genotype–environment association was found by the Wald test, and results for all other variables were identified by the G‐test. GWAS results affected by linkage (*n* = 99) are marked in Table S2. With the exception of five SNPs (identified in Table S4), the following results include only loci unaffected by linkage.

We examined each SNP and one megabase (Mb; one million bases) on either side (hereafter flanking regions) in the UCSC dog genome browser (http://genome.ucsc.edu/cgi-bin/hgTracks) and the NCBI Map Viewer (http://www.ncbi.nlm.nih.gov/projects/mapview/) to identify genes or genomic regions known or assumed to be of functional importance (henceforth referred to as functional genes). Thirty‐two key functional genes (or groups of genes) near SNP loci identified as outliers (*n* = 27) and/or associated with environmental variables (*n* = 22) are listed in Table 2 and divided into groups based on function: temperature (*n* = 3), metabolism (*n* = 9), and physical development (*n* = 20). One SNP was associated with variables (latitude, annual temperature) found to be correlated (Table 2; Table S1). Complete nomenclature and identification for SNP loci are provided in Table S2. Furthermore, we observed SNPs near key functional genes associated with features for which we do not have environmental data or that appear important to all wolves across their range. We have highlighted *n* = 12 SNPs associated with disease and parasites, *n* = 16 for sensory functions, and *n* = 9 for brain and cognition (Table S3). Four of these SNPs were associated with correlated variables (Tables S1 and S3).

**Table 2:** Functional genes near single nucleotide polymorphisms (SNP) identified as outlier loci and/or associated with environmental variables based on a study of 59 wolves in four European population clusters. Environmental variables are given in Table 1. Full locus identification from the Illumina CanineHD BeadChip is provided in Table S2. Function summary is based on references from the NCBI database (http://www.ncbi.nlm.nih.gov/gene)

Chr and SNP number^a^	BayeScan log10(PO)^b^	BayeScan FDR^c^	SAM result^d^	*F*_ST_ e	Gene(s)	Function summary
						TEMPERATURE
Chr9_143	0.904 (4P) 1.134 (BU)	0.058 (4P) 0.034 (BU)	–	0.327	RPTOR	Thermogenesis
Chr9_148	1.217 (4P)	0.027 (4P)	jult (AA)	0.332	TRPV1/TRPV3	Thermoregulation
Chr25_269	0.771 (BC)	0.069 (BC)	bioc (AA)	0.197	TRPM8	Thermosensation (cold sensor)
						METABOLISM
Chr5_85	–	–	bioc (GA)	0.022	SGIP1	Fat mass, food intake
Chr5_85	–	–	bioc (GA)	0.022	LEPR	Fat metabolism
Chr5_100	0.860 (CU)	0.079 (CU)	bioc (AC)	0.260	TK2	mtDNA synthesis
Chr9_151	–	–	bioc (AA,CC)	0.236	CRAT	Energy homeostasis, fat metabolism
Chr9_151	–	–	bioc (AA,CC)	0.236	DNM1	Exercise‐induced collapse
Chr15_188	1.089 (4P)	0.034 (4P)	bioc (GG)	0.230	NPYR1	Vasoconstriction in exercising skeletal muscle
Chr18_208	0.725 (4P) 1.118 (NC)	0.080 (4P) 0.064 (NC)	bioc (AG,GG)	0.202	CPT1A	mtDNA membrane, lipid metabolism
Chr26_280	0.813 (NU)	0.068 (NU)	bioc (AA), jult (GG)	0.255	SLC5A1	Carbohydrate digestion/absorption.
Chr32_326	–	–	bioc (CG)	0.033	SCD5	Energy metabolism
						PHYSICAL DEVELOPMENT
Chr3_23	0.841 (4P) 1.341 (BC)	0.067 (4P) 0.028 (BC)	–	0.342	IGFI1R	Reduced size (dogs)
Chr4_46	0.889 (4P) 1.423 (NB)	0.059 (4P) 0.017 (NB)	lat, alt (AA)	0.497	ZFR	RNA regulation
Chr13_169	1.207 (4P) 1.214 (CU)	0.028 (4P) 0.042 (CU)	–	0.260	RSPO2	Dog coat color
Chr13_175	1.329 (CU)	0.038 (CU)	–	0.283	KIT	Dog coat patterns (spotted Weimaraner)
Chr15_183	0.511 (NB)	0.098 (NB)	–	0.231	ATP2B1	Intracellular calcium homeostasis; vascular smooth muscle cells; possibly Chagas disease (American trypanosomiasis)
Chr15_187	1.168 (4P) 1.648 (NU)	0.030 (4P) 0.015 (NU)	–	0.332	FNIP2	Hypomyelination in the brain; spinal cord defects (Weimaraner dogs)
Chr18_208	0.725 (4P) 1.118 (NC)	0.080 (4P) 0.064 (NC)	bioc (AG,GG)	0.202	FGF4	Bone morphogenesis
Chr19_210	1.206 (NU)	0.033 (NU)	–	0.303	DARS	Hypomyelination (brain, spinal cord)
Chr21_217	0.591 (4P) 1.475 (NB) 0.672 (BU)	0.096 (4P) 0.014 (NB) 0.096 (BU)	–	0.288	PPFIBP2	Neural synapse development
Chr21_222	0.629 (NU)	0.111 (NU)	bioc (AG,GG)	0.231	HPS5	Hermansky–Pudlak syndrome (oculocutaneous albinism, platelet abnormality)
Chr21_223	0.818 (NU)	0.061 (NU)	lat, annt (AA)^f^	0.395	NAV2	Neuron growth and regeneration
Chr21_225	0.804 (NU)	0.074 (NU)	–	0.226	ANO3	Dominant craniocervical dystonia (sustained muscle contractions – repetitive movements or abnormal postures); eczema, asthma
Chr23_236	0.908 (4P) 0.794 (NU)	0.052 (4P) 0.085 (NU)	prec (AG,GG)	0.361	AGTR1	Angiotensin II (blood pressure and volume)
Chr23_236	0.908 (4P) 0.794 (NU)	0.052 (4P) 0.085 (NU)	prec (AG,GG)	0.361	HPS3	Hermansky–Pudlak syndrome (oculocutaneous albinism, platelet abnormality)
Chr23_236	0.908 (4P) 0.794 (NU)	0.052 (4P) 0.085 (NU)	prec (AG,GG)	0.361	CP	Aceruloplasminemia (iron accumulation and tissue damage)
Chr24_245	0.537 (4P)	0.106 (4P)	long (AA)	0.340	BMP7	Bone growth
Chr24_246	0.579 (NB)	0.079 (NB)	bioc (GG, GA, AA)	0.338	COL9A3	Collagen (dwarfism, ocular defects)
Chr26_281	1.547 (4P) 0.779 (NU)	0.012 (4P) 0.089 (NU)	bioc (GG)	0.352	ADORA2A	Cardiac rhythm and circulation, blood flow, immune function, pain regulation, sleep
Chr28_299	1.1880 (NB)	0.008 (NB)	lat (GG)	0.290	SPRCS3	Central nervous system development
Chr31_317	1.062 (BC)	0.043 (BC)	bioc (GG)	0.315	ADAMTS1	Organ morphology and function

a Full SNP identification given in Table S2.

b Pairwise comparisons for: B – Balkan‐Dinaric; C – Carpathian Mountains.; U – Ukrainian Steppe; N – northcentral Europe. 4P: across all four clusters.

c False discovery rate threshold (q‐value).

d Environmental variables identified by the spatial analysis method (SAM) as significantly associated with one or more genotypes. SAM incorporates two separate tests: the Wald and the likelihood ratio (G) test (Joost et al. 2007). The variable “bioc” was identified by the Wald test; all other variables by the G‐test. No result was identified in both.

e *F*_ST_ calculated across all 353 loci for all population clusters.

f Correlations between (some) variables. See Table S1 with results for all variable combinations.

**Figure 3:**
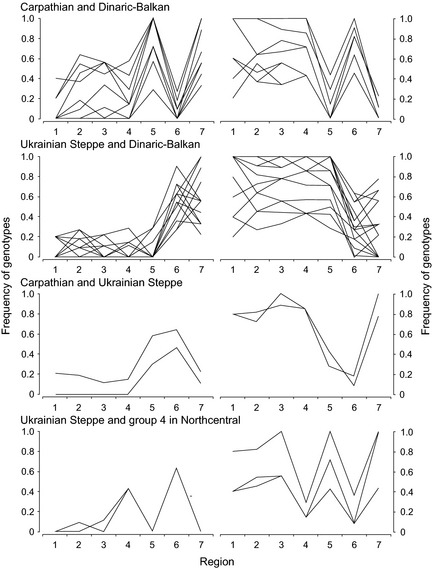
Spatial distributions of European wolf single nucleotide polymorphism (SNP) loci/genotypes typical for two neighboring clusters. The graphs show frequencies of loci/genotypes differentiating among wolves in northcentral (groups 1–4), Carpathian (5), Ukrainian Steppe (6), and Dinaric‐Balkan clusters (group 7). Numbers on *x*‐axis are wolf groups 1–7 (see Fig. 1). Left panels: loci/genotypes with high frequencies in the two clusters. Right panels: loci/genotypes with low frequencies in the two clusters. SNP loci and genotypes are listed in Table S4.

Our results exhibited clear spatial patterns in one (Figs. 1, 2) or – less frequently – two population clusters (Fig. 3), including SNPs near genes for functions believed to be important for wolves across their range. Certain of these SNPs showed distinct geographic distributions of genotypes (Table S4). For example, genotypes varied between the Carpathian Mountains and the Ukrainian Steppe/Dinaric‐Balkan clusters for SNP Chr13_175 located near the gene KIT (dog coat pattern). We selected one representative genotype for each cluster for interpolation into continuous surface (Fig. 1). We then noted SNPs near genes for important functions that showed no obvious spatial patterns (Table S5). Several SNPs were also identified as strong outliers in BayeScan but not associated with any of the 12 environmental variables, nor were there any functional genes reported in the 1‐Mb flanking regions. These results might nevertheless be of interest for future investigation (Table S6).

**Figure 4:**
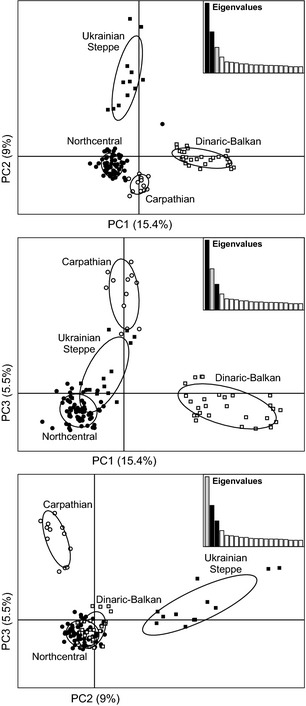
Principal component analyses results for *n* = 113 European wolves in 4 population clusters with 353 loci. Population clusters: northcentral Europe (*n* = 60), Carpathian Mountains (*n* = 12), Ukrainian Steppe (*n* = 12), Dinaric‐Balkan (*n* = 29). Upper panel: PC axes 1 and 2. Middle panel: PC axes 1 and 3. Lower panel: PC axes 2 and 3. Percentages of variation explained by PC1–PC3 shown on axes.

Pairwise *F*_ST_ values between the four population clusters, with the full sample of 113 individuals and all 353 loci, showed the highest value for Carpathian Mountains – Ukrainian Steppe – and the lowest value for Carpathian Mountains – northcentral Europe (Table 3). Pairwise *F*_ST_ values for the sample of 59 individuals were similarly high and generally consistent with the larger sample, although the highest value was between northcentral Europe and Dinaric‐Balkan and the lowest was for northcentral Europe – Ukrainian Steppe (Table S7). Principal component analyses of all 113 wolves showed differentiation among all population clusters (Fig. 4). Although northcentral Europe and Carpathian Mountain individuals overlapped on the 1st axis, they were clearly distinct on the 3rd axis. The 1st axis reflects north–south differentiation in European wolves, whereas the 2nd axis generally (although there is some spatial overlap between northcentral Europe and Ukrainian Steppe) indicates east–west structure. When compared to the other three clusters, the individual profiles from northcentral Europe appear highly concentrated relative to their spatial distribution (Figs. 1, 4).

**Table 3:** Pairwise *F*_ST_ values with 95% confidence intervals for *n* = 113 wolves in four population cluster, across *n* = 353 SNP loci reported as outliers (BayeScan) or associated with environmental variables (GWAS in PLINK), calculated in HierFstat with bootstrap resampling (*n* = 1000). All were significant at *P* < 0.001 except^a^

Cluster (*n*)	Northcentral Europe (*n* = 60)	Ukrainian Steppe (*n* = 12)	Dinaric‐Balkan (*n* = 29)
Ukrainian Steppe (*n* = 12)	0.220 [0.191–0.246]	–	–
Dinaric‐Balkan (*n* = 29)	0.227 [0.196–0.257]	0.240 [0.213–0.269]	–
Carpathian Mountains (*n* = 12)	0.190 [0.155–0.226]	0.243^a^ [0.212–0.274]	0.223 [0.187–0.256]

a *P* = 0.013.

## Discussion

Our results identified genes potentially influencing local adaptation for temperature, metabolism, physical development, and disease/immune system functions in European wolves. However, the importance of SNPs associated with genes for putative local adaptations appears overshadowed by findings linked to traits of universal importance, including hearing, vision, olfaction, and cognitive functions. This suggests that ancient, concurrent, and possibly parallel selection may have played a more prominent role than recent local adaptation in structuring functional genetic variation in wolves throughout our study area. Concurrent selection for ubiquitous traits in separate populations may have been divergent or parallel. However, for traits such as hearing and vision a trajectory of parallel selection appears most likely.

Our results nonetheless suggest local adaptation may play a role. Although the wolf is a highly mobile species, it has been reported to exhibit population structure corresponding with environmental heterogeneity in Europe (Pilot et al. 2006, 2012) and North America (Geffen et al. 2004; Musiani et al. 2007; Muñoz‐Fuentes et al. 2009; Stronen et al. 2014). Our findings indicate that variables such as temperature and habitat may influence local adaptation, which appears consistent with earlier results from the study area (Pilot et al. 2006). A SNP flanking two genes reported to influence temperature regulation (TRPV1/TRPV3) was associated with July temperature. Because wolves are long‐distance pursuing (as opposed to ambush) predators, physiological mechanisms to prevent overheating could represent important selective factors. The possibility of local environmental selection for temperature regulation merits further investigation, particularly in light of warming earth surface temperatures and changes in the degree of variability for temperature and other climatic factors.

Local adaptation can occur if individuals are more likely to survive and reproduce within their natal habitats (Davis and Stamps 2004; Nosil et al. 2005; Edelaar et al. 2008), which subsequently affects population genetic structure. The wolf population clusters examined in this study (Stronen et al. 2013) are exposed to markedly different climatic factors such as temperature and precipitation. Northcentral European and Carpathian wolves are not usually subject to very hot weather but experience cold (including subzero) temperatures extensive parts of the year, whereas the opposite is typically true for the Balkan‐Dinaric wolves of southern Europe. Ukrainian Steppe wolves, in contrast, may experience both hot summers and cold winters. Although speculative, the capacity for temperature regulation might play a particularly important role for wolves in the steppe. The northcentral and Carpathian environments have much in common with regard to climate. The differences in day length between the two areas likely influence other processes of ecological importance such as plant photoperiods, and differences in day length have been reported to affect the behavior of Arctic mammals such as Svalbard reindeer (*R. t. platyrhynchus*) (van Oort et al. 2005). The clear structuring seen between Carpathian and northcentral European wolves, which reflects the division into two major phylogenetic clades of wolves (Pilot et al. 2010; Czarnomska et al. 2013), could, at least in part, also be caused by habitat fragmentation and human landscape development (Huck et al. 2011).

The divergent profiles of Ukrainian Steppe wolves may to some extent be a result of immigration from outside the study area. The Ukrainian part of our study area could be receiving immigrants from the steppe or forest‐steppe regions farther east and north, and similar immigration from eastern and northern regions may occur in the western Russian part of our study area (Pilot 2005). *F*_ST_ values for 67‐K loci were lowest between northcentral Europe and Ukrainian Steppe wolves (Stronen et al. 2013). Drift is expected to influence the entire genome and selection to act only on certain loci, and the *F*_ST_ values for the 353 loci between northcentral Europe and the Ukrainian Steppe suggest diversifying selection might play a role in increasing divergence between wolves from these regions. Spatio‐temporal resolution of the selective forces that may have produced the current patterns is challenging because of limited available data from the eastern part of our study area and beyond – for our study and in general.

Prey and habitat have been reported as important variables in earlier investigations with (presumed) neutral markers (Geffen et al. 2004; Musiani et al. 2007; Muñoz‐Fuentes et al. 2009; Stronen et al. 2014). This includes findings from our study area (Pilot et al. 2006, 2012). Importantly, neither ecosystem nor biome may be the appropriate scale at which to examine the local patterns of selection in species such as wolves; ecosystem may be too narrow, whereas biome could be too broad. Other features of the local environment, such as the size and behavior of available prey, may be more informative for elucidating the patterns of selection (Benson et al. 2012; Monzon et al. 2014). We did not have prey data for our study area, but earlier investigations within Europe (Jędrzejewski et al. 2012; Pilot et al. 2012, 2014a) accord with new data from North America (Benson et al. 2012; Monzon et al. 2014) in suggesting that the influence of diet merits further attention.

None of the BayeScan and GWAS candidate loci overlapped, although a number of SAM results were supported by outlier detection as well as gene–environment associations. Earlier studies have reported similar lack of overlap between BayeScan and other tests (e.g., Narum and Hess 2011). Several potentially important drivers of selection are not included in our study (e.g., diet, disease, and parasites), which might help explain why a number of outlier loci in BayeScan were not identified in gene–environment tests. However, we would expect loci detected by environmental selection to be identified by BayeScan, and it is uncertain why this did not occur. Possibly, methodical differences may play a role. For example, Bayesian methods implemented in BayeScan differ from that of significance testing in classical statistics (Foll 2012). Another factor could be our small sample sizes from some population clusters. We included a large number of loci per individual, but certain tests might require more samples to identify important genotypes and alleles. The possible presence of polygenic effects – small changes in allele frequencies at a large number of loci (Pritchard and Di Rienzo 2010) – combined with relatively strict correction for multiple testing (Joost et al. 2007) could also help explain the discrepancies between the test results. Finally, GWAS in PLINK and SAM are individual‐based whereas BayeScan examines populations, which could mask important heterogeneity.

All our BayeScan results indicated directional selection, yet balancing selection has been reported for MHC loci in wolves from Finland (Niskanen et al. 2014). Whereas outlier methods appear prone to reporting false positives for balancing selection (Narum and Hess 2011), we cannot exclude the possibility of balancing selection in our data set that we were unable to detect. We identified certain loci associated with the MHC complex, but our study did not include any data on diseases or parasites. The absence of signals for balancing selection in our study might reflect our choice of variables and limitations of BayeScan combined with strict criteria for avoiding false positives.

The spatio‐temporal presence of glacial refugia for wolves is not resolved, and contributions from possible glacial refugia in eastern Europe and Asia may be undervalued (Taberlet et al. 1998). Furthermore, the range of large northern wolves could have been restricted during interglacial periods during which these wolves appear to have been replaced by smaller forms (Kahlke 1999). Such a scenario might produce a complex spatio‐temporal genetic admixture that confounds the effects of repeated recolonization events with the presence of locally adapted ecotypes. Besides, the strength and direction of selective pressures may have varied with time (Coop et al. 2009). A recent study including profiles from Caucasian wolves suggested gene flow between Caucasus and eastern Europe (Pilot et al. 2014b), and additional genomic investigation of samples from far eastern Europe and Asia is an important priority.

A historic wolf ecomorph specializing in megafaunal prey appears to have gone extinct in North America (Leonard et al. 2007). This mtDNA haplogroup (henceforth haplogroup 2) was common in ancient European wolves, but seems to have been replaced over much of the continent and is now largely confined to southern Europe (Pilot et al. 2010). The reason(s) for the apparent replacement is uncertain, and larger prey species such as moose and reindeer are more common in northern and central Europe (Sidorovich et al. 2003; Kojola et al. 2004; Jędrzejewski et al. 2010), whereas prey species in southern areas are typically small to mid‐size (Papageorgiou et al. 1994; Kusak et al. 2005). The relationship between the “megafaunal” mtDNA haplogroup 2 and the current wolf of southern Europe therefore merits additional attention. Possibly, mtDNA haplogroup 2 persists in wolves now adapted to the warmer climate and smaller prey species of the contemporary environment.

We observed a number of SNPs near genes coding for traits that seem important for wolves across their range, including memory and olfaction. These results exhibited spatial patterns consistent with previously observed population clusters. In humans, recent reports imply that selection on different genes involved in, for example, immunity can occur in separate populations (Laurent et al. 2012). We hypothesize that the high prevalence of SNPs flanking genes associated with universal traits may, at least in part, reflect parallel evolution. We recommend additional research, with numerically and geographically larger sample sizes, to explore whether differentiation linked to such ubiquitous traits might indicate deep evolutionary divergence among population clusters.

### Main limitations of the study

No SAM result was detected in the cumulative test that requires significance for both Wald and G‐tests, which might suggest that our results are not robust. However, the Wald test shows a lack of power for smaller sample sizes (Quinn and Keough 2002). The Bonferroni correction implies a very conservative test level (in this case, an adjusted *P*‐level of 3.93e‐06) that should yield only the most robust associations (Joost et al. 2007). Pilot et al. (2006) found temperature to be important in explaining neutral genetic structure within our study area. As the area extends from arctic tundra to the Mediterranean Sea, with large variations in summer and winter temperatures, our findings appear to offer a reasonable explanation.

Although we accepted levels of relatedness up to 0.1, which might have included distantly related individuals, the PLINK calculation of relatedness assumes a relatively homogeneous sample. Because of population structure in European wolves (Stronen et al. 2013), our results for pairwise relatedness are expected to be conservative as samples within one cluster will appear relatively more similar to each other than to wolves from other clusters. Yet, European wolves have been found to disperse >800 km (Wabakken et al. 2007; Andersen et al. 2015), and because of uncertainties associated with gaps in our sampling (e.g., whether wolf profiles from Romania might represent a cline between Carpathian and Dinaric‐Balkan clusters, Stronen et al. 2013 Fig. 1), we preferred to screen for relatedness across the entire sample. We are therefore confident that relatedness will not have confounded our results.

Our study is descriptive (nonexperimental), and we cannot exclude the possible influence of other evolutionary factors such as genetic drift. Wolves are apex predators with large home ranges (Jędrzejewski et al. 2007) and occur at low density on the landscape. Despite their high dispersal capability, a history of small and fragmented wolf populations in large parts of Europe (Linnell et al. 2008) has probably caused substantial amounts of genetic drift because of population fluctuations and bottlenecks, as well as family structure and low effective population size (Mech and Boitani 2003).

Another possible confounding factor may be the presence of endogenous incompatibilities that exhibit spatial structuring in accordance with environmental transition zones and therefore (incorrectly) suggest that selection is driven by environmental factors (Bierne et al. 2011). Although we examined over 67‐K SNPs in a well‐studied species with a wide global distribution, it remains challenging unequivocally to separate influences of selection, endogenous incompatibilities, and genetic drift, and the results must be interpreted with caution. Furthermore, it can be difficult to determine whether genes are under selection or rather extreme outliers in the neutral distribution (Coop et al. 2009). Our findings for, for example, metabolism and temperature regulation might also involve pleiotropic effects whereby one gene exerts influence on multiple apparently unrelated traits (Lynch and Hayden 1995). Further attention is also warranted on the importance of polygenic traits, which can be difficult to identify with common tools for detecting selection because of small changes at multiple loci (Pritchard and Di Rienzo 2010 and references therein). We could have implemented alternate multiple‐test procedures such as the false discovery rate correction instead of the more stringent Bonferroni adjustment to augment the chance of detecting such loci. However, we opted to focus on the more robust SNP results and flanking genes under (possible) selection in our study area.

## Conclusion

Our findings suggest that ancient, concurrent, and possibly parallel selection could have played a more prominent role than recent local adaptation in structuring functional genetic variation in wolves throughout our study area. However, local adaptation may also have influenced the population structure and evolution of European wolves. The results indicate differences between northern and southern Europe, the Carpathian Mountain and Ukrainian Steppe population clusters for a number of SNP loci and neighboring genes with known or assumed functions. Although several genes appear to be associated with environmental features, others, such as genes implicated in olfaction, are presumably important for all wolves and may reflect parallel selection. Several strong outlier loci, many of which contributed clearly to population structure, were not associated with any of 12 environmental factors under study. Although genetic drift and small population size complicate the ability to evaluate local adaptation in wolves, the species is a well‐suited study organism because of its adaptability (as evidenced by its broad global distribution) and capacity for long‐distance movement. Future research could moreover illuminate how wild species such as wolves might adapt to their local environments by means of hybridization with related species, which represents a rapid means of acquiring genetic variation that has already been filtered through natural selection. We examined contemporary individuals, but results will indicate selective pressures as they occurred in the past. Additional attention is required to understand how wild organisms will respond to ongoing or future influences such as climate change and shifts in the distribution and abundance of the species that constitute their main diet.

## Data accessibility

Sampling locations and single nucleotide polymorphism genotypes: Dryad doi: 10.5061/dryad.p6598.

## Conflict of Interest

None declared.

## Supporting information


**Table S1.** Correlation between environmental variables (detailed in Table 1).
**Table S2.** Complete identification for single nucleotide polymorphism (SNP) loci on the Illumina CanineHD BeadChip (170K SNPs) with information from the MAP‐file in PLINK.
**Table S3.** Summary of major functional genes near single nucleotide polymorphism (SNP) loci identified as outlier loci and/or associated with environmental variables based on a study of 59 wolves in four European population clusters.
**Table S4.** Functional genes where genotype frequencies show spatial patterns between population clusters (e.g., Northcentral against the other three, or Northcentral and Ukrainian Steppe against others).
**Table S5.** Functional genes without obvious spatial patterns.
**Table S6.** SNP loci identified as outliers by BayeScan but not associated with environmental variables included in this study.
**Table S7.** Pairwise *F*_ST_‐values with 95% confidence intervals for *n* = 59 wolves in four population cluster, across *n* = 353 SNP loci reported as outliers (BayeScan) or associated with environmental variables (GWAS in PLINK), calculated in HierFstat with bootstrap resampling (*n* = 1000).

## References

[ece31695-bib-0001] Andersen, L. W. , V. Harms , R. Caniglia , S. D. Czarnomska , E. Fabbri , B. Jezdrzejewska , et al. 2015 Long‐distance dispersal of a wolf, *Canis lupus*, in Northwestern Europe. Mamm. Res. 60:163–168.

[ece31695-bib-0002] André, C. , L. C. Larsson , L. Laikre , D. Bekkevold , J. Brigham , G. R. Carvalho , et al. 2011 Detecting population structure in a high gene‐flow species, Atlantic herring (*Clupea harengus*): direct, simultaneous evaluation of neutral vs. putatively selected loci. Heredity 106:270–280. 20551979 10.1038/hdy.2010.71 PMC3183876

[ece31695-bib-0003] Balloux, F. , and N. Lugon‐Moulin . 2002 The estimation of population differentiation with microsatellite markers. Mol. Ecol. 11:155–165. 11856418 10.1046/j.0962-1083.2001.01436.x

[ece31695-bib-0004] Benson, J. F. , B. R. Patterson , and T. J. Wheeldon . 2012 Spatial genetic and morphologic structure of wolves and coyotes in relation to environmental heterogeneity in a Canis hybrid zone. Mol. Ecol. 21:5934–5954. 23173981 10.1111/mec.12045

[ece31695-bib-0005] Bierne, N. , J. Welch , E. Loire , F. Bonhomme , and P. David . 2011 The coupling hypothesis: why genome scans may fail to map local adaptation genes. Mol. Ecol. 20:2044–2072. 21476991 10.1111/j.1365-294X.2011.05080.x

[ece31695-bib-0006] Bowen, B. W. , and S. A. Karl . 2007 Population genetics and phylogeography of sea turtles. Mol. Ecol. 16:4886–4907. 17944856 10.1111/j.1365-294X.2007.03542.x

[ece31695-bib-0007] Carmichael, L. E. , J. A. Nagy , N. C. Larter , and C. Strobeck . 2001 Prey specialization may influence patterns of gene flow in wolves of the Canadian Northwest. Mol. Ecol. 10:2787–2798. 11903892 10.1046/j.0962-1083.2001.01408.x

[ece31695-bib-0008] Coop, G. , J. K. Pickrell , J. Novembre , S. Kudaravalli , J. Li , D. Absher , et al. 2009 The role of geography in human adaptation. PLoS Genet. 5:e1000500. 19503611 10.1371/journal.pgen.1000500 PMC2685456

[ece31695-bib-0009] Czarnomska, S. D. , B. Jędrzejewska , T. Borowik , M. Niedziałkowska , A. V. Stronen , S. Nowak , et al. 2013 Concordant mitochondrial and microsatellite DNA structuring between Polish lowland and Carpathian wolves. Conserv. Genet. 14:573–588.

[ece31695-bib-0010] Davis, J. M. , and J. A. Stamps . 2004 The effect of natal experience on habitat preferences. Trends Ecol. Evol. 19:411–416. 16701298 10.1016/j.tree.2004.04.006

[ece31695-bib-0011] De Souza, C. L. Jr , I. O. Geraldi , and R. Vencovsky . 2000 Response to recurrent selection under small effective population size. Genet. Mol. Biol. 23:841–846.

[ece31695-bib-0012] DeFaveri, J. , P. R. Jonsson , and J. Merila . 2013 Heterogeneous genomic differentiation in marine threespine sticklebacks: adaptation along an environmental gradient. Evolution 67:2530–2546. 24033165 10.1111/evo.12097

[ece31695-bib-0013] Edelaar, P. , A. M. Siepielski , and J. Clobert . 2008 Matching habitat choice causes directed gene flow: a neglected dimension in evolution and ecology. Evolution 62:2462–2472. 18637835 10.1111/j.1558-5646.2008.00459.x

[ece31695-bib-0014] ESRI . 2013 ArcGIS desktop: release 10.2. Environmental Systems Research Institute, Redlands, CA.

[ece31695-bib-0015] Fabbri, E. , C. Miquel , V. Lucchini , A. Santini , R. Caniglia , C. Duchamp , et al. 2007 From the Apennines to the Alps: colonization genetics of the naturally expanding Italian wolf (*Canis lupus*) population. Mol. Ecol. 16:1661–1671. 17402981 10.1111/j.1365-294X.2007.03262.x

[ece31695-bib-0016] Foll, M. 2012 BayeScan v2.1 User Manual (from http://cmpg.unibe.ch/software/BayeScan/), 10 pp.

[ece31695-bib-0017] Foll, M. , and O. Gaggiotti . 2008 A genome‐scan method to identify selected loci appropriate for both dominant and codominant markers: a Bayesian perspective. Genetics 180:977–993. 18780740 10.1534/genetics.108.092221 PMC2567396

[ece31695-bib-0018] Freedman, M. L. , D. Reich , K. L. Penney , G. J. McDonald , A. A. Mignault , N. Patterson , et al. 2004 Assessing the impact of population stratification on genetic association studies. Nat. Genet. 36:388–393. 15052270 10.1038/ng1333

[ece31695-bib-0019] Geffen, E. , M. J. Anderson , and R. K. Wayne . 2004 Climate and habitat barriers to dispersal in the highly mobile grey wolf. Mol. Ecol. 13:2481–2490. 15245420 10.1111/j.1365-294X.2004.02244.x

[ece31695-bib-0020] Goudet, J. 2005 HierFstat, a package for R to compute and test hierarchical F‐statistics. Mol. Ecol. Notes 5:184–186.

[ece31695-bib-0021] Hammer, Ø. , D. A. T. Harper , and P. D. Ryan . 2001 PAST: Paleontological statistics software package for education and data analysis. Palaeontol. Electronica 4:9.

[ece31695-bib-0022] Hoelzel, A. R. , J. Hey , M. E. Dahlheim , C. Nicholson , V. Burkanov , and N. Black . 2007 Evolution of population structure in a highly social top predator, the killer whale. Mol. Biol. Evol. 24:1407–1415. 17400573 10.1093/molbev/msm063

[ece31695-bib-0023] Huck, M. , W. Jędrzejewski , T. Borowik , B. Jędrzejewska , S. Nowak , and R. W. Mysłajek . 2011 Analyses of least cost path for determining effects of habitat types on landscape permeability: wolves in Poland. Acta Theriol. 56:91–101. 21350594 10.1007/s13364-010-0006-9 PMC3026926

[ece31695-bib-0024] Jędrzejewski, W. , B. Jędrzejewska , Ž. Andersone‐Lilley , L. Balčiauskas , P. Männil , and J. Ozolins , et al. (2010) Synthesizing wolf ecology and management in eastern Europe: similarities and contrasts with North America Pp. 207–233 *in* Musiani M., Boitani L., Paquet P. C., eds. The world of wolves: new perspectives on ecology, behaviour and management. University of Calgary Press, Calgary, Canada.

[ece31695-bib-0025] Jędrzejewski, W. , K. Schmidt , J. Theuerkauf , B. Jędrzejewska , and R. Kowalczyk . 2007 Territory size of wolves *Canis lupus*: linking local (Białowieża Primeval Forest, Poland) and Holarctic‐scale patterns. Ecography 30:66–76.

[ece31695-bib-0026] Jędrzejewski, W. , M. Niedziałkowska , M. W. Hayward , J. Goszczyński , B. Jędrzejewska , T. Borowik , et al. 2012 Prey choice and diet of wolves related to ungulate communities and wolf subpopulations in Poland. J. Mammal. 93:1480–1492.

[ece31695-bib-0027] Jombart, T. 2008 adegenet: a R package for the multivariate analysis of genetic markers. Bioinformatics 24:1403–1405. 18397895 10.1093/bioinformatics/btn129

[ece31695-bib-0028] Joost, S. , and M. Kalbermatten . 2010 MatSAM version 2 beta, 26 pp (from http://www.econogene.eu/software/sam/).

[ece31695-bib-0029] Joost, S. , A. Bonin , M. W. Bruford , L. Després , C. Conord , G. Erhardt , et al. 2007 A spatial analysis method (SAM) to detect candidate loci for selection: towards a landscape genomics approach to adaptation. Mol. Ecol. 16:3955–3969. 17850556 10.1111/j.1365-294X.2007.03442.x

[ece31695-bib-0030] Joost, S. , M. Kalbermatten , and A. Bonin . 2008 Spatial analysis method (SAM): a software tool combining molecular and environmental data to identify candidate loci for selection. Mol. Ecol. Resour. 8:957–960. 21585940 10.1111/j.1755-0998.2008.02162.x

[ece31695-bib-0031] Kahlke, R.‐D. 1999 The History of the origin, evolution and dispersal of the Late Pleistocene Mammuthus‐Coelodonta faunal complex in Eurasia (Large Mammals). Mammoth Site of Hot Springs, South Dakota.

[ece31695-bib-0032] Kojola, I. , O. Huitu , K. Toppinen , K. Heikura , S. Heikkinen , and S. Ronkainen . 2004 Predation on European wild forest reindeer (*Rangifer tarandus*) by wolves (*Canis lupus*) in Finland. J. Zool. 263:229–235.

[ece31695-bib-0033] Kusak, J. , A. Majić Skrbinšek , and D. Huber . 2005 Home ranges, movements, and activity of wolves (*Canis lupus*) in the Dalmatian part of Dinarids, Croatia. Eur. J. Wildl. Res. 51:254–262.

[ece31695-bib-0034] Laurent, R. , B. Toupance , and R. Chaix . 2012 Non‐random mate choice in humans: insights from a genome scan. Mol. Ecol. 21:587–596. 22121833 10.1111/j.1365-294X.2011.05376.x

[ece31695-bib-0035] Legendre, P. , and L. Legendre . 1998 Numerical ecology, 2nd ed Elsevier, Amsterdam, the Netherlands.

[ece31695-bib-0036] Leonard, J. A. , C. Vila , K. Fox‐Dobbs , P. L. Koch , R. K. Wayne , and B. Van Valkenburgh . 2007 Megafaunal extinctions and the disappearance of a specialized wolf ecomorph. Curr. Biol. 17:1146–1150. 17583509 10.1016/j.cub.2007.05.072

[ece31695-bib-0037] Linnell, J. , V. Salvatori , and L. Boitani . 2008 Guidelines for population level management plans for large carnivores in Europe. A Large Carnivore Initiative for Europe report prepared for the European Commission (contract 070501/2005/424162/MAR/B2).

[ece31695-bib-0038] Lucchini, V. , A. Galov , and E. Randi . 2004 Evidence of genetic distinction and long‐term population decline in wolves (*Canis lupus*) in the Italian Apennines. Mol. Ecol. 13:523–536. 14871358 10.1046/j.1365-294x.2004.02077.x

[ece31695-bib-0039] Lynch, J. M. , and J. M. Hayden . 1995 Genetic influences on cranial form: variation among ranch and feral American mink Mustela vison (Mammalia: Mustelidae). Biol. J. Linn. Soc. 55:293–307.

[ece31695-bib-0040] Manel, S. , E. Bellemain , J. E. Swenson , and O. Francois . 2004 Assumed and inferred spatial structure of populations: the Scandinavian brown bears revisited. Mol. Ecol. 13:1327–1331. 15078468 10.1111/j.1365-294X.2004.02074.x

[ece31695-bib-0041] McRae, B. H. , P. Beier , L. E. Dewald , L. Y. Huynh , and P. Keim . 2005 Habitat barriers limit gene flow and illuminate historical events in a wide‐ranging carnivore, the American puma. Mol. Ecol. 14:1965–1977. 15910319 10.1111/j.1365-294x.2005.02571.x

[ece31695-bib-0042] Mech, L. D. , and L. Boitani (2003) Wolf social ecology Pp. 1–34 *in* Mech L. D., Boitani L., eds. Wolves: behaviour, ecology and conservation. University of Chicago Press, Chicago, IL.

[ece31695-bib-0043] Milano, I. , M. Babbucci , A. Cariani , M. Atanassova , D. Bekkevold , G. R. Carvalho , et al. 2014 Outlier SNP markers reveal fine‐scale genetic structuring across European hake populations (*Merluccius merluccius*). Mol. Ecol. 23:118–135. 24138219 10.1111/mec.12568

[ece31695-bib-0044] Monzon, J. , R. Kays , and D. E. Dykhuizen . 2014 Assessment of coyote‐wolf‐dog admixture using ancestry‐informative diagnostic SNPs. Mol. Ecol. 23:182–197. 24148003 10.1111/mec.12570 PMC3899836

[ece31695-bib-0045] Muñoz‐Fuentes, V. , C. T. Darimont , R. K. Wayne , P. C. Paquet , and J. A. Leonard . 2009 Ecological factors drive differentiation in wolves from British Columbia. J. Biogeogr. 36:1516–1531.

[ece31695-bib-0046] Musiani, M. , J. A. Leonard , H. D. Cluff , C. C. Gates , S. Mariani , P. C. Paquet , et al. 2007 Differentiation of tundra/taiga and boreal coniferous forest wolves: genetics, coat colour and association with migratory caribou. Mol. Ecol. 16:4149–4170. 17725575 10.1111/j.1365-294X.2007.03458.x

[ece31695-bib-0047] Narum, S. R. , and J. E. Hess . 2011 Comparison of F(ST) outlier tests for SNP loci under selection. Mol. Ecol. Resour. 11(Suppl. 1):184–194. 21429174 10.1111/j.1755-0998.2011.02987.x

[ece31695-bib-0048] Niskanen, A. K. , L. J. Kennedy , M. Ruokonen , I. Kojola , H. Lohi , M. Isomursu , et al. 2014 Balancing selection and heterozygote advantage in major histocompatibility complex loci of the bottlenecked Finnish wolf population. Mol. Ecol. 23:875–889. 24382313 10.1111/mec.12647

[ece31695-bib-0049] Nosil, P. , T. H. Vines , and D. J. Funk . 2005 Perspective: reproductive isolation caused by natural selection against immigrants from divergent habitats. Evolution 59:705–719. 15926683

[ece31695-bib-0050] van Oort, B. E. H. , N. J. C. Tyler , M. P. Gerkema , L. Folkow , A. Schytte Blix , and K.‐A. Stokkan . 2005 Circadian organization in reindeer. Nature 438:1095–1096. 16371996 10.1038/4381095a

[ece31695-bib-0051] Papageorgiou, N. , C. Vlachos , A. Sfougaris , and E. Tsachalidis . 1994 Status and diet of wolves in Greece. Acta Theriol. 39:411–416.

[ece31695-bib-0052] Pariset, L. , S. Joost , P. A. Marsan , A. Valentini , and Econogene Consortium (EC) . 2009 Landscape genomics and biased *F* _ST_ approaches reveal single nucleotide polymorphisms under selection in goat breeds of North‐East Mediterranean. BMC Genet. 10:7. 19228375 10.1186/1471-2156-10-7 PMC2663570

[ece31695-bib-0053] Pertoldi, C. , R. Bijlsma , and V. Loeschcke . 2007 Conservation genetics in a globally changing environment: present problems, paradoxes and future challenges. Biodivers. Conserv. 16:4147–4163.

[ece31695-bib-0054] Pilot, M. 2005 Genetic variability and population structure of wolf Canis lupus in central and eastern Europe. PhD Dissertation, Museum and Institute of Zoology, Polish Academy of Sciences, Warsaw, Poland.

[ece31695-bib-0055] Pilot, M. , W. Jędrzejewski , W. Branicki , V. E. Sidorovich , B. Jędrzejewska , and K. Stachura , et al. 2006 Ecological factors influence population genetic structure of European grey wolves. Mol. Ecol. 15:4533–4553. 17107481 10.1111/j.1365-294X.2006.03110.x

[ece31695-bib-0056] Pilot, M. , W. Branicki , W. Jędrzejewski , J. Goszczyński , B. Jędrzejewska , I. Dykyy , et al. 2010 Phylogeographic history of grey wolves in Europe. BMC Evol. Biol., 10:104. 20409299 10.1186/1471-2148-10-104 PMC2873414

[ece31695-bib-0057] Pilot, M. , W. Jędrzejewska , V. E. Sidorovich , W. Meier‐Augenstein , and A. R. Hoelzel . 2012 Dietary differentiation and the evolution of population genetic structure in a highly mobile carnivore. PLoS One 7:e39341. 22768075 10.1371/journal.pone.0039341 PMC3387138

[ece31695-bib-0058] Pilot, M. , C. Greco , B. M. vonHoldt , B. Jędrzejewska , E. Randi , W. Jędrzejewska , et al. 2014a Genome‐wide signatures of population bottlenecks and diversifying selection in European wolves. Heredity 112:428–442. 24346500 10.1038/hdy.2013.122 PMC3966127

[ece31695-bib-0059] Pilot, M. , M. J. Dąbrowski , V. Hayrapetyan , E. G. Yavruyan , N. Kopaliani , E. Tsingarska , et al. 2014b Genetic variability of the grey wolf *Canis lupus* in the Caucasus in comparison with Europe and the Middle East: distinct or intermediary population? PLoS One 9:e93828. 24714198 10.1371/journal.pone.0093828 PMC3979716

[ece31695-bib-0060] Pritchard, J. K. , and A. Di Rienzo . 2010 Adaptation ‐ not by sweeps alone. Nat. Rev. Genet. 11:665–667. 20838407 10.1038/nrg2880 PMC4652788

[ece31695-bib-0061] Purcell, S. , B. Neale , K. Todd‐Brown , L. Thomas , M. A. Ferreira , D. Bender , et al. 2007 PLINK: a tool set for whole‐genome association and population‐based linkage analyses. Am. J. Hum. Genet. 81:559–575. 17701901 10.1086/519795 PMC1950838

[ece31695-bib-0062] Quinn, G. P. , and M. J. Keough . 2002 Experimental design and data analysis for biologists. Cambridge Univ. Press, Cambridge, U.K.

[ece31695-bib-0063] R Development Core Team 2012 R: A language and environment for statistical computing. R Foundation for Statistical Computing, Vienna, Austria.

[ece31695-bib-0064] Randi, E. 2011 Genetics and conservation of wolves *Canis lupus* in Europe. Mamm. Rev. 41:99–111.

[ece31695-bib-0065] Rousset, F. 2008 GENEPOP ‘007: a complete reimplementation of the GENEPOP software for Windows and Linux. Mol. Ecol. Resour. 8:103–106. 21585727 10.1111/j.1471-8286.2007.01931.x

[ece31695-bib-0066] Rueness, E. K. , N. C. Stenseth , M. O'Donoghue , S. Boutin , H. Ellegren , and K. S. Jakobsen . 2003 Ecological and genetic spatial structuring in the *Canadian lynx* . Nature 425:69–72. 12955141 10.1038/nature01942

[ece31695-bib-0067] Sacks, B. N. , S. K. Brown , and H. B. Ernest . 2004 Population structure of California coyotes corresponds to habitat‐specific breaks and illuminates species history. Mol. Ecol. 13:1265–1275. 15078462 10.1111/j.1365-294X.2004.02110.x

[ece31695-bib-0068] Sacks, B. N. , B. R. Mitchell , C. L. Williams , and H. B. Ernest . 2005 Coyote movements and social structure along a cryptic population genetic subdivision. Mol. Ecol. 14:1241–1249. 15773950 10.1111/j.1365-294X.2005.02473.x

[ece31695-bib-0069] Sidorovich, V. E. , L. L. Tikhomirova , and B. Jędrzejewska . 2003 Wolf *Canis lupus* numbers, diet and damage to livestock in relation to hunting and ungulate abundance in northeastern Belarus during 1990–2000. Wildl. Biol. 9:103–111.

[ece31695-bib-0070] Slatkin, M. 1985 Gene flow in natural populations. Annu. Rev. Ecol. Syst. 16:393–430.

[ece31695-bib-0071] Slatkin, M. 1987 Gene flow and the geographic structure of natural populations. Science 236:787–792. 3576198 10.1126/science.3576198

[ece31695-bib-0072] Smith, E. N. , W. Chen , M. Kahonen , M. Kähönen , J. Kettunen , T. Lehtimäki , L. Peltonen , et al. 2010 Longitudinal genome‐wide association of cardiovascular disease risk factors in the Bogalusa heart study. PLoS Genet. 6:e1001094. 20838585 10.1371/journal.pgen.1001094 PMC2936521

[ece31695-bib-0073] Sommer, R. , and N. Benecke . 2005 Late‐Pleistocene and early Holocene history of canid fauna of Europe (Canidae). Mamm. Biol. 70:227–241.

[ece31695-bib-0074] Sommer, R. S. , and A. Nadachowski . 2006 Glacial refugia of mammals in Europe: evidence from fossil records. Mamm. Rev. 36:251–265.

[ece31695-bib-0075] Stronen, A. V. , B. Jedrzejewska , C. Pertoldi , D. Demontis , E. Randi , M. Niedzia?kowska , et al. 2013 North‐South differentiation and a region of high diversity in European wolves (*Canis lupus*). PLoS One 8:e76454. 24146871 10.1371/journal.pone.0076454 PMC3795770

[ece31695-bib-0076] Stronen, A. V. , E. L. Navid , M. S. Quinn , P. C. Paquet , H. M. Bryan , and C. T. Darimont . 2014 Population genetic structure of gray wolves (*Canis lupus*) in a marine archipelago suggests island‐mainland differentiation consistent with dietary niche. BMC Ecol., 14:11. 24915756 10.1186/1472-6785-14-11 PMC4050401

[ece31695-bib-0077] Taberlet, P. , L. Fumagalli , A. G. Wust‐Saucy , and J. F. Cosson . 1998 Comparative phylogeography and postglacial colonization routes in Europe. Mol. Ecol. 7:453–464. 9628000 10.1046/j.1365-294x.1998.00289.x

[ece31695-bib-0078] Wabakken, P. , H. Sand , I. Kojola , B. Zimmermann , J. M. Arnemo , H. C. Pedersen , et al. 2007 Multistage, long‐range natal dispersal by a global positioning system‐collared Scandinavian wolf. J. Wildl. Manage. 71:1631–1634.

[ece31695-bib-0079] Weckworth, B. V. , S. Talbot , G. K. Sage , D. K. Person , and J. Cook . 2005 A signal for independent coastal and continental histories among North American wolves. Mol. Ecol. 14:917–931. 15773925 10.1111/j.1365-294X.2005.02461.x

[ece31695-bib-0080] Weckworth, B. V. , S. L. Talbot , and J. A. Cook . 2010 Phylogeography of wolves (*Canis lupus*) in the Pacific Northwest. J. Mammal. 91:363–375.

[ece31695-bib-0081] Weckworth, B. V. , N. G. Dawson , S. L. Talbot , M. J. Flamme , and J. A. Cook . 2011 Going coastal: shared evolutionary history between coastal British Columbia and Southeast Alaska wolves (*Canis lupus*). PLoS One 6:e19582. 21573241 10.1371/journal.pone.0019582 PMC3087762

