# Supplementary material for: Genome‐wide analyses suggest parallel selection for universal traits may eclipse local environmental selection in a highly mobile carnivore
Source: Ecol Evol. 2015 Sep 22;5(19):4410–25. doi: 10.1002/ece3.1695 (PMC4667828; doi:10.1002/ece3.1695)
Supplement: Supplementary file 1 — Table S1. Correlation between environmental variables (detailed in Table 1). Table S2. Complete identification for single nucleotide polymorphism (SNP) loci on the Illumina CanineHD BeadChip (170K SNPs) with information from the MAP‐file in PLINK. Table S3. Summary of major functional genes near single nucleotide polymorphism (SNP) loci identified as outlier loci and/or associated with environmental variables based on a study of 59 wolves in four European population clusters. Table S4. Functional genes where genotype frequencies show spatial patterns between population clusters (e.g., Northcentral against the other three, or Northcentral and Ukrainian Steppe against others). Table S5. Functional genes without obvious spatial patterns. Table S6. SNP loci identified as outliers by BayeScan but not associated with environmental variables included in this study. Table S7. Pairwise F ST‐values with 95% confidence intervals for n = 59 wolves in four population cluster, across n = 353 SNP loci reported as outliers (BayeScan) or associated with environmental variables (GWAS in PLINK), calculated in HierFstat with bootstrap resampling (n = 1000). [file ECE3-5-4410-s001.docx]

**Supplemental Materials for the article “Genome-wide analyses suggest parallel selection for universal traits may eclipse local environmental selection in a highly mobile carnivore” (Stronen et al. 2015, Ecology and Evolution doi: 10.1002/ece3.1695)**

Table S1. Correlation between environmental variables (detailed in Table 1). Values below the diagonal show the non-parametric Kendall’s rank correlation coefficient (Kendall’s tau). Values above the diagonal show tests of significance.

|  | **long** | **lat** | **popd** | **ecoc** | **bioc** | **annt** | **jant** | **jult** | **prec** | **road** | **alt** | **snow** |
| --- | --- | --- | --- | --- | --- | --- | --- | --- | --- | --- | --- | --- |
| **long** | -- | 0.299 | 0.197 | 0.047 | 0.069 | 0.091 | 0.000* | 0.084 | 0.000* | 0.000* | 0.969 | 0.001 |
| **lat** | 0.093 | -- | 0.000* | 0.255 | 0.000* | 0.000* | 0.000* | 0.000* | 0.722 | 0.002 | 0.020 | 0.000* |
| **popd** | -0.115 | -0.473 | -- | 0.021 | 0.016 | 0.000* | 0.000* | 0.000* | 0.510 | 0.001 | 0.027 | 0.000* |
| **ecoc** | 0.178 | 0.102 | -0.206 | -- | 0.003 | 0.744 | 0.749 | 0.447 | 0.966 | 0.677 | 0.000* | 0.321 |
| **bioc** | 0.163 | -0.348 | 0.215 | -0.267 | -- | 0.013 | 0.202 | 0.033 | 0.049 | 0.186 | 0.045 | 0.407 |
| **annt** | -0.151 | **-0.624** | 0.365 | 0.029 | 0.222 | -- | 0.000* | 0.000* | 0.116 | 0.004 | 0.126 | 0.000* |
| **jant** | -0.420 | -0.545 | 0.335 | -0.029 | 0.114 | **0.656** | -- | 0.000* | 0.198 | 0.000* | 0.252 | 0.000* |
| **jult** | 0.154 | -0.508 | 0.339 | 0.068 | 0.190 | **0.698** | 0.347 | -- | 0.000* | 0.711 | 0.049 | 0.000* |
| **prec** | -0.496 | 0.032 | -0.059 | 0.004 | -0.176 | -0.141 | 0.115 | -0.372 | -- | 0.005 | 0.364 | 0.635 |
| **road** | -0.477 | -0.279 | 0.287 | -0.037 | -0.118 | 0.255 | 0.469 | 0.033 | 0.249 | -- | 0.361 | 0.000* |
| **alt** | 0.004 | -0.208 | 0.197 | -0.313 | 0.179 | -0.137 | -0.102 | -0.176 | 0.081 | 0.082 | -- | 0.011 |
| **snow** | 0.294 | 0.383 | -0.340 | 0.089 | -0.074 | **-0.626** | **-0.651** | -0.442 | 0.042 | -0.312 | 0.228 | -- |

Bold font – high correlation (> 0.6)

Underscore – moderate correlation (0.3 - 0.6)

Normal font – uncorrelated (< 0.3)

* significant at p = 0.05 after Bonferroni correction

Table S2. Complete identification for single nucleotide polymorphism (SNP) loci on the Illumina CanineHD BeadChip (170K SNPs) with information from the MAP-file in PLINK^1^. SNP number refers to the codes assigned in the evaluation of 353 loci identified by genome-wide association study (GWAS) and BayeScan. This code is used as a short-hand locus identifier when presenting and discussing the results. Loci marked with (*) were removed during LD-pruning.

| **Chromosome** | **Chromosome & SNP number** | **SNP identifier** | **Genetic distance (morgans)** | **Base-pair position (bp units)** |
| --- | --- | --- | --- | --- |
| 1 | Chr1_1 | BICF2P463407 | 21,3034 | 12068463 |
| 1 | Chr1_2 | BICF2G630713680 | 38,4563 | 20254729 |
| 1 | Chr1_3 | TIGRP2P17376_rs8899111 | 44,7865 | 25755827 |
| 1 | Chr1_4 | BICF2S23223100 | 56,0124 | 35035028 |
| 1 | *Chr1_5 | BICF2G630721326 | 63,1902 | 39915037 |
| 1 | Chr1_6 | BICF2P684995 | 84,387 | 59536208 |
| 1 | Chr1_7 | BICF2S23225902 | 138,544 | 116864229 |
| 1 | Chr1_8 | BICF2P962032 | 141,114 | 122192287 |
| 1 | Chr1_9 | BICF2S23218065 | 141,247 | 125191714 |
| 1 | Chr1_10 | BICF2S23642042 | 141,254 | 125357488 |
| 2 | Chr2_11 | BICF2P831241 | 34,7552 | 14676428 |
| 2 | Chr2_12 | BICF2S23547745 | 39,96 | 17388904 |
| 2 | Chr2_13 | BICF2P431443 | 46,3543 | 22818420 |
| 2 | Chr2_14 | BICF2P1412676 | 48,342 | 25664924 |
| 2 | *Chr2_15 | BICF2P1042369 | 49,4525 | 26295720 |
| 2 | Chr2_16 | BICF2P521676 | 59,1119 | 35346623 |
| 2 | Chr2_17 | BICF2P1441095 | 66,3194 | 42103436 |
| 2 | Chr2_18 | BICF2G630494785 | 88,1182 | 66367147 |
| 2 | Chr2_19 | BICF2P1260990 | 92,8396 | 70088605 |
| 3 | Chr3_20 | TIGRP2P36496_rs9179969 | 13,0615 | 4138894 |
| 3 | Chr3_21 | TIGRP2P36590_rs8840854 | 15,0041 | 4871621 |
| 3 | Chr3_22 | BICF2P460360 | 53,2166 | 29480798 |
| 3 | Chr3_23 | BICF2G630340944 | 69,4707 | 45920551 |
| 3 | Chr3_24 | BICF2P777990 | 70,89 | 49660497 |
| 3 | Chr3_25 | BICF2S23333400 | 86,8039 | 63341213 |
| 3 | *Chr3_26 | BICF2G630344913 | 92,9257 | 67686137 |
| 3 | Chr3_27 | BICF2P959251 | 93,1289 | 68053465 |
| 3 | Chr3_28 | BICF2G630346294 | 94,7235 | 70352777 |
| 3 | Chr3_29 | BICF2S23731276 | 101,485 | 73560324 |
| 3 | *Chr3_30 | BICF2S22958901 | 102,884 | 74397432 |
| 3 | Chr3_31 | BICF2G630358530 | 110,143 | 91201876 |
| 3 | Chr3_32 | TIGRP2P56697_rs8554529 | 110,272 | 93668058 |
| 4 | Chr4_33 | BICF2P728 | 26,032 | 12509890 |
| 4 | Chr4_34 | BICF2S23230859 | 30,3334 | 15636451 |
| 4 | Chr4_35 | BICF2P870454 | 51,3044 | 31012655 |
| 4 | Chr4_36 | BICF2P442956 | 52,92 | 32098934 |
| 4 | Chr4_37 | BICF2P347472 | 53,4485 | 33695021 |
| 4 | Chr4_38 | TIGRP2P60724_rs8628257 | 65,8327 | 46572600 |
| 4 | Chr4_39 | BICF2P1275210 | 69,4949 | 55633534 |
| 4 | Chr4_40 | BICF2S23549200 | 70,06 | 55988281 |
| 4 | Chr4_41 | BICF2S23641432 | 70,2267 | 56092934 |
| 4 | Chr4_42 | BICF2S23023204 | 71,577 | 56940642 |
| 4 | Chr4_43 | BICF2P719540 | 72,9999 | 57689270 |
| 4 | Chr4_44 | BICF2P747023 | 78,8248 | 66856348 |
| 4 | Chr4_45 | BICF2P1357850 | 82,4734 | 73002031 |
| 4 | Chr4_46 | G315f33S162 | 86,4485 | 78255358 |
| 4 | *Chr4_47 | BICF2S23746430 | 87,6404 | 79273252 |
| 4 | *Chr4_48 | BICF2G630170710 | 87,6496 | 79292734 |
| 4 | *Chr4_49 | BICF2G630172911 | 88,781 | 81570824 |
| 4 | *Chr4_50 | BICF2G630175659 | 91,1647 | 84563038 |
| 4 | *Chr4_51 | BICF2G630177564 | 93,3857 | 86642118 |
| 4 | *Chr4_52 | BICF2G630178419 | 94,317 | 87198946 |
| 4 | *Chr4_53 | BICF2P723899 | 95,5003 | 88862713 |
| 5 | Chr5_54 | BICF2G63032418 | 48,3308 | 29625075 |
| 5 | *Chr5_55 | BICF2G630182589 | 56,3195 | 34523422 |
| 5 | *Chr5_56 | BICF2G630182632 | 56,3503 | 34569605 |
| 5 | *Chr5_57 | BICF2S23631833 | 56,861 | 34879011 |
| 5 | *Chr5_58 | BICF2G630182807 | 56,9097 | 34955867 |
| 5 | *Chr5_59 | BICF2G630182831 | 56,9252 | 34980333 |
| 5 | *Chr5_60 | BICF2G630182835 | 56,9307 | 34988366 |
| 5 | *Chr5_61 | BICF2G630182839 | 56,9459 | 34998591 |
| 5 | *Chr5_62 | BICF2G630182847 | 56,9641 | 35010844 |
| 5 | *Chr5_63 | BICF2G630182861 | 56,9758 | 35018779 |
| 5 | *Chr5_64 | BICF2G630182874 | 56,9918 | 35029528 |
| 5 | Chr5_65 | BICF2G630182886 | 57,0319 | 35056533 |
| 5 | *Chr5_66 | BICF2G630182901 | 57,109 | 35108429 |
| 5 | *Chr5_67 | BICF2G630182902 | 57,1125 | 35110780 |
| 5 | *Chr5_68 | BICF2S23652976 | 57,2508 | 35203942 |
| 5 | *Chr5_69 | BICF2P621135 | 57,6492 | 35496567 |
| 5 | *Chr5_70 | BICF2G630183158 | 57,8025 | 36129586 |
| 5 | *Chr5_71 | BICF2G630183333 | 58,1787 | 36380114 |
| 5 | *Chr5_72 | BICF2S23438844 | 58,9834 | 37128092 |
| 5 | *Chr5_73 | BICF2P68050 | 58,987 | 37134649 |
| 5 | *Chr5_74 | BICF2P252322 | 59,0374 | 37225409 |
| 5 | *Chr5_75 | BICF2G630184335 | 61,2862 | 38298615 |
| 5 | *Chr5_76 | BICF2G630184645 | 62,4772 | 38758791 |
| 5 | *Chr5_77 | BICF2G630184875 | 63,8734 | 39298279 |
| 5 | *Chr5_78 | BICF2G630185003 | 64,5213 | 39629615 |
| 5 | *Chr5_79 | BICF2G630185015 | 64,5461 | 39661283 |
| 5 | *Chr5_80 | BICF2G630185640 | 64,9283 | 40758915 |
| 5 | *Chr5_81 | BICF2P682357 | 65,0356 | 40965077 |
| 5 | *Chr5_82 | BICF2P937318 | 67,0364 | 45196265 |
| 5 | *Chr5_83 | BICF2S23018364 | 67,0945 | 45312776 |
| 5 | *Chr5_84 | BICF2P1375880 | 67,2855 | 46181807 |
| 5 | Chr5_85 | BICF2G630187117 | 67,3016 | 46785340 |
| 5 | *Chr5_86 | BICF2P250380 | 67,3158 | 47317049 |
| 5 | *Chr5_87 | TIGRP2P71570_rs9248163 | 67,3173 | 47372989 |
| 5 | *Chr5_88 | BICF2G630187815 | 67,3365 | 48090358 |
| 5 | *Chr5_89 | BICF2P334730 | 67,3408 | 48250241 |
| 5 | *Chr5_90 | BICF2G630188374 | 67,3664 | 49207892 |
| 5 | Chr5_91 | BICF2P855616 | 67,3746 | 49513375 |
| 5 | Chr5_92 | BICF2G630188963 | 67,3926 | 50187749 |
| 5 | Chr5_93 | BICF2P786597 | 67,41 | 50914929 |
| 5 | Chr5_94 | BICF2P1006725 | 67,7483 | 51798403 |
| 5 | *Chr5_95 | BICF2G630189772 | 67,8005 | 51876778 |
| 5 | Chr5_96 | BICF2P1324481 | 69,0186 | 53364577 |
| 5 | Chr5_97 | BICF2P857994 | 69,1 | 53435286 |
| 5 | Chr5_98 | TIGRP2P73280_rs8716956 | 70,0838 | 54293460 |
| 5 | *Chr5_99 | BICF2P1185405 | 71,1011 | 55513332 |
| 5 | Chr5_100 | TIGRP2P75235_rs8884972 | 101,882 | 85738342 |
| 6 | Chr6_101 | BICF2G630807896 | 14,0068 | 5311154 |
| 6 | *Chr6_102 | BICF2P658867 | 15,1626 | 5749209 |
| 6 | *Chr6_103 | BICF2P1005381 | 15,18 | 5779207 |
| 6 | *Chr6_104 | BICF2P939836 | 15,18 | 5843844 |
| 6 | *Chr6_105 | BICF2P415895 | 15,5262 | 6099202 |
| 6 | *Chr6_106 | BICF2S23050935 | 15,5489 | 6107984 |
| 6 | *Chr6_107 | BICF2P785778 | 16,0649 | 6307446 |
| 6 | *Chr6_108 | TIGRP2P85377_rs9069041 | 16,2543 | 6380698 |
| 6 | *Chr6_109 | BICF2P1367401 | 16,3677 | 6424507 |
| 6 | *Chr6_110 | BICF2S23214236 | 17,7406 | 6804191 |
| 6 | *Chr6_111 | BICF2G630808440 | 19,8621 | 7822527 |
| 6 | *Chr6_112 | BICF2P794739 | 21,0187 | 8596608 |
| 6 | Chr6_113 | BICF2P732609 | 31,4092 | 14187655 |
| 6 | Chr6_114 | TIGRP2P79322_rs8867438 | 35,4552 | 16289391 |
| 6 | *Chr6_115 | BICF2S23759798 | 35,8827 | 16468281 |
| 6 | *Chr6_116 | BICF2G630165660 | 37,2808 | 16875303 |
| 6 | *Chr6_117 | BICF2P199049 | 39,1114 | 17923386 |
| 6 | *Chr6_118 | BICF2G630166022 | 39,2152 | 17978221 |
| 6 | *Chr6_119 | BICF2P641063 | 40,6675 | 18929706 |
| 6 | Chr6_120 | BICF2P1249700 | 58,536 | 39455548 |
| 6 | Chr6_121 | BICF2P885681 | 60,7992 | 40814424 |
| 6 | Chr6_122 | BICF2S2316381 | 72,1 | 46994759 |
| 6 | Chr6_123 | BICF2P281040 | 80,6723 | 63287011 |
| 6 | Chr6_124 | BICF2G630572879 | 83,08 | 68370518 |
| 6 | Chr6_125 | BICF2P1167880 | 83,7027 | 69612597 |
| 6 | Chr6_126 | BICF2P492151 | 87,9688 | 74665975 |
| 6 | Chr6_127 | BICF2P223115 | 90,6541 | 80621071 |
| 7 | Chr7_128 | BICF2P555908 | 17,1792 | 8596555 |
| 7 | Chr7_129 | BICF2G630552738 | 32,2657 | 18284512 |
| 7 | Chr7_130 | BICF2P1465743 | 72,1054 | 50211422 |
| 7 | Chr7_131 | BICF2S2361703 | 75,9935 | 54513734 |
| 7 | Chr7_132 | BICF2P745035 | 76,0424 | 54643121 |
| 7 | Chr7_133 | BICF2G630560987 | 78,49 | 60402322 |
| 7 | Chr7_134 | BICF2G630565370 | 79,0066 | 66453165 |
| 7 | Chr7_135 | BICF2P914492 | 79,6915 | 67266225 |
| 7 | Chr7_136 | BICF2G63084362 | 82,741 | 69920643 |
| 7 | Chr7_137 | BICF2G63085688 | 86,5369 | 73499402 |
| 8 | *Chr8_138 | TIGRP2P116578_rs8603887 | 19,0421 | 7780762 |
| 8 | *Chr8_139 | BICF2S23528499 | 23,4221 | 12364082 |
| 8 | Chr8_140 | BICF2G630413714 | 71,8929 | 60313100 |
| 8 | Chr8_141 | BICF2P568648 | 76,01 | 65872906 |
| 8 | Chr8_142 | BICF2P771892 | 78,3791 | 69225467 |
| 9 | Chr9_143 | BICF2S2319928 | 8,10809 | 4078769 |
| 9 | Chr9_144 | BICF2P1007553 | 24,3646 | 11096033 |
| 9 | Chr9_145 | BICF2P939832 | 49,1487 | 26713984 |
| 9 | *Chr9_146 | BICF2P425977 | 52,7203 | 28351844 |
| 9 | Chr9_147 | BICF2G630832428 | 53,1443 | 29312147 |
| 9 | Chr9_148 | BICF2P328665 | 64,2263 | 49931564 |
| 9 | Chr9_149 | BICF2P728721 | 64,2375 | 50204714 |
| 9 | Chr9_150 | BICF2S22931940 | 64,3017 | 51776397 |
| 9 | Chr9_151 | BICF2G630473547 | 64,5512 | 57882238 |
| 10 | Chr10_152 | BICF2P555215 | 25,8346 | 9547807 |
| 10 | Chr10_153 | BICF2P569451 | 60,0065 | 32074999 |
| 10 | *Chr10_154 | BICF2P534526 | 62,33 | 36094752 |
| 10 | Chr10_155 | BICF2G630487152 | 63,82 | 41498001 |
| 10 | Chr10_156 | TIGRP2P140015_rs8854829 | 66,5703 | 46368121 |
| 10 | Chr10_157 | BICF2S23625084 | 72,7183 | 54591875 |
| 11 | Chr11_158 | BICF2P1395717 | 40,0938 | 24105860 |
| 12 | Chr12_159 | BICF2P1458084 | 13,5181 | 4556823 |
| 12 | *Chr12_160 | BICF2P681456 | 25,5594 | 11227831 |
| 12 | Chr12_161 | BICF2P464391 | 70,4562 | 53794952 |
| 12 | Chr12_162 | BICF2S23234469 | 72,4265 | 55448431 |
| 12 | Chr12_163 | BICF2S24313073 | 74,8357 | 60338364 |
| 12 | Chr12_164 | BICF2G630120578 | 76,0484 | 61471390 |
| 12 | Chr12_165 | BICF2P409406 | 80,462 | 65094089 |
| 12 | Chr12_166 | TIGRP2P170770_rs8762531 | 81,1453 | 65550229 |
| 12 | Chr12_167 | BICF2P1013041 | 85,7502 | 69756381 |
| 13 | Chr13_168 | BICF2G630602219 | 0 | 3174563 |
| 13 | Chr13_169 | BICF2S23223177 | 0 | 10644572 |
| 13 | Chr13_170 | BICF2P424681 | 0 | 13287200 |
| 13 | Chr13_171 | BICF2G630606320 | 0 | 13294754 |
| 13 | Chr13_172 | BICF2P760434 | 0 | 17554950 |
| 13 | Chr13_173 | BICF2G630610292 | 0 | 19741416 |
| 13 | Chr13_174 | BICF2P696842 | 33,2565 | 34133087 |
| 13 | Chr13_175 | BICF2S23218616 | 52,84 | 49420761 |
| 13 | Chr13_176 | BICF2G630750329 | 58,7035 | 61077563 |
| 13 | Chr13_177 | BICF2G630747649 | 59,13 | 63423944 |
| 14 | Chr14_178 | BICF2P1374301 | 37,5369 | 33982585 |
| 14 | Chr14_179 | BICF2P410268 | 37,7078 | 34055491 |
| 14 | Chr14_180 | TIGRP2P193626_rs9073696 | 45,42 | 46698513 |
| 14 | Chr14_181 | BICF2P274339 | 45,42 | 46831256 |
| 14 | Chr14_182 | BICF2S22946866 | 60,4367 | 60308645 |
| 15 | Chr15_183 | BICF2G630432576 | 33,4003 | 33594671 |
| 15 | *Chr15_184 | TIGRP2P203488_rs8885535 | 52,8238 | 52983214 |
| 15 | Chr15_185 | TIGRP2P205385_rs8969615 | 59,7032 | 57617904 |
| 15 | *Chr15_186 | BICF2G630424162 | 59,7941 | 57672865 |
| 15 | Chr15_187 | BICF2G630423543 | 60,3657 | 58244369 |
| 15 | Chr15_188 | BICF2G630419038 | 66,3593 | 63377242 |
| 16 | Chr16_189 | BICF2P1191049 | 56,9014 | 32535959 |
| 16 | Chr16_190 | BICF2G630112997 | 57,0106 | 33995246 |
| 16 | Chr16_191 | BICF2P733388 | 57,3045 | 37923776 |
| 16 | Chr16_192 | BICF2P1333553 | 57,3218 | 38156278 |
| 16 | Chr16_193 | BICF2P1074838 | 57,3964 | 39153082 |
| 16 | Chr16_194 | TIGRP2P215553_rs8903479 | 57,5166 | 40759636 |
| 16 | Chr16_195 | BICF2S2292520 | 57,5622 | 41368407 |
| 16 | Chr16_196 | BICF2G630820664 | 76,7463 | 57604994 |
| 17 | Chr17_197 | BICF2P450900 | 13,2626 | 5304170 |
| 17 | Chr17_198 | BICF2P556210 | 14,2948 | 5661003 |
| 17 | *Chr17_199 | BICF2P917040 | 23,4606 | 8791916 |
| 17 | Chr17_200 | BICF2S2452036 | 49,2148 | 19933506 |
| 17 | Chr17_201 | BICF2S23018034 | 50,6191 | 21874860 |
| 17 | Chr17_202 | BICF2P228747 | 78,793 | 47207600 |
| 17 | Chr17_203 | BICF2P1020659 | 78,8175 | 47219526 |
| 17 | Chr17_204 | BICF2G630284611 | 89,5975 | 55925364 |
| 17 | Chr17_205 | BICF2P288535 | 90,8334 | 57145053 |
| 17 | Chr17_206 | BICF2S23239183 | 100,822 | 65030832 |
| 18 | Chr18_207 | BICF2G630690944 | 74,7591 | 48592877 |
| 18 | Chr18_208 | BICF2P787912 | 76,1111 | 50845034 |
| 19 | Chr19_209 | BICF2P871165 | 25,7295 | 8078265 |
| 19 | Chr19_210 | BICF2S2304227 | 63,187 | 41733351 |
| 19 | Chr19_211 | BICF2P874955 | 81,0299 | 54531223 |
| 20 | Chr20_212 | BICF2S23427244 | 55,2729 | 29644658 |
| 20 | Chr20_213 | BICF2P572457 | 55,4961 | 30166526 |
| 20 | Chr20_214 | BICF2S23640344 | 92,1741 | 54779660 |
| 21 | Chr21_215 | BICF2P323445 | 10,0438 | 16589686 |
| 21 | Chr21_216 | BICF2P993447 | 12,4899 | 17979000 |
| 21 | Chr21_217 | BICF2P1156772 | 38,7897 | 34412279 |
| 21 | Chr21_218 | BICF2P1055206 | 41,7868 | 35523232 |
| 21 | Chr21_219 | BICF2P410928 | 41,865 | 35552227 |
| 21 | Chr21_220 | BICF2P678289 | 45,2072 | 37835971 |
| 21 | Chr21_221 | TIGRP2P285659_rs9253593 | 64,2538 | 43862563 |
| 21 | Chr21_222 | BICF2P1384440 | 64,2555 | 43937982 |
| 21 | Chr21_223 | TIGRP2P286158_rs8510310 | 68,7828 | 45011618 |
| 21 | Chr21_224 | BICF2S22963884 | 77,4714 | 46670111 |
| 21 | Chr21_225 | BICF2G630640337 | 98,006 | 50589795 |
| 22 | Chr22_226 | BICF2G630315933 | 0 | 7447647 |
| 22 | Chr22_227 | BICF2G630316477 | 0 | 9371657 |
| 22 | *Chr22_228 | BICF2P997358 | 38,2812 | 32485402 |
| 22 | Chr22_229 | BICF2S23436608 | 54,9253 | 43171779 |
| 22 | Chr22_230 | BICF2P661890 | 109,954 | 58018905 |
| 22 | Chr22_231 | BICF2P153993 | 127,53 | 62390482 |
| 22 | Chr22_232 | BICF2P1423684 | 128,306 | 62583530 |
| 23 | Chr23_233 | BICF2P18072 | 0 | 37580324 |
| 23 | Chr23_234 | BICF2G630370840 | 0 | 39523054 |
| 23 | Chr23_235 | BICF2P329457 | 0 | 44331177 |
| 23 | Chr23_236 | BICF2P87666 | 0 | 47364324 |
| 23 | Chr23_237 | BICF2G630366534 | 0 | 50462296 |
| 23 | *Chr23_238 | BICF2G630364841 | 0 | 51901147 |
| 24 | Chr24_239 | BICF2G630511547 | 0 | 12267546 |
| 24 | Chr24_240 | BICF2G630506560 | 0 | 18304649 |
| 24 | Chr24_241 | BICF2G630505097 | 0 | 20378668 |
| 24 | Chr24_242 | TIGRP2P312306_rs8836249 | 0 | 20591622 |
| 24 | Chr24_243 | BICF2P1103677 | 0 | 24161779 |
| 24 | Chr24_244 | BICF2P967247 | 0 | 37821273 |
| 24 | Chr24_245 | BICF2G630497792 | 0 | 44256952 |
| 24 | Chr24_246 | BICF2G630495908 | 0 | 49809134 |
| 25 | *Chr25_247 | BICF2P756236 | 0 | 19791602 |
| 25 | *Chr25_248 | TIGRP2P325963_rs9165179 | 0 | 20144057 |
| 25 | *Chr25_249 | BICF2P1266815 | 0 | 20872498 |
| 25 | *Chr25_250 | BICF2P466539 | 0 | 22014726 |
| 25 | *Chr25_251 | BICF2P462950 | 0 | 22445841 |
| 25 | Chr25_252 | TIGRP2P326785_rs8509320 | 0 | 23667228 |
| 25 | Chr25_253 | BICF2G63094181 | 0 | 23772340 |
| 25 | Chr25_254 | BICF2S23414927 | 0 | 24340132 |
| 25 | *Chr25_255 | BICF2S236314 | 0 | 24364469 |
| 25 | *Chr25_256 | BICF2P604554 | 0 | 24755010 |
| 25 | *Chr25_257 | BICF2P299246 | 0 | 26577794 |
| 25 | *Chr25_258 | BICF2S23056261 | 0 | 26779863 |
| 25 | *Chr25_259 | BICF2S2317316 | 0 | 27557971 |
| 25 | *Chr25_260 | BICF2G63094740 | 0 | 28276084 |
| 25 | *Chr25_261 | BICF2P1209962 | 0 | 29110586 |
| 25 | *Chr25_262 | BICF2G63095195 | 0 | 29321329 |
| 25 | Chr25_263 | BICF2G63096215 | 0 | 30511067 |
| 25 | Chr25_264 | BICF2P1151598 | 0 | 31159042 |
| 25 | *Chr25_265 | BICF2G63097157 | 0 | 31760309 |
| 25 | *Chr25_266 | BICF2P1127880 | 0 | 35668862 |
| 25 | Chr25_267 | BICF2S23413966 | 0 | 37278827 |
| 25 | Chr25_268 | BICF2S23345522 | 0 | 46607965 |
| 25 | Chr25_269 | BICF2S23519543 | 0 | 48436684 |
| 25 | Chr25_270 | TIGRP2P333972_rs8758243 | 0 | 51208992 |
| 25 | Chr25_271 | BICF2G630158020 | 0 | 52108271 |
| 26 | Chr26_272 | BICF2P720436 | 0 | 3977899 |
| 26 | Chr26_273 | BICF2P758169 | 0 | 4025325 |
| 26 | Chr26_274 | BICF2P191851 | 0 | 6089085 |
| 26 | Chr26_275 | BICF2P53749 | 0 | 15344205 |
| 26 | Chr26_276 | BICF2G630802966 | 0 | 17699972 |
| 26 | Chr26_277 | BICF2G630802320 | 0 | 18440920 |
| 26 | Chr26_278 | BICF2P735774 | 0 | 20173488 |
| 26 | Chr26_279 | BICF2G630798809 | 0 | 23219238 |
| 26 | Chr26_280 | BICF2S24114718 | 0 | 27970526 |
| 26 | Chr26_281 | BICF2S23151747 | 0 | 31738914 |
| 26 | Chr26_282 | BICF2P854140 | 0 | 37477078 |
| 26 | *Chr26_283 | TIGRP2P344506_rs9138824 | 0 | 38035539 |
| 27 | Chr27_284 | BICF2G630145506 | 0 | 18679709 |
| 27 | Chr27_285 | BICF2P151318 | 0 | 20976253 |
| 27 | Chr27_286 | BICF2P27556 | 0 | 25195575 |
| 27 | Chr27_287 | BICF2P893308 | 0 | 37791819 |
| 27 | Chr27_288 | BICF2S23535135 | 0 | 37814333 |
| 27 | Chr27_289 | TIGRP2P354702_rs8810091 | 0 | 38184004 |
| 27 | Chr27_290 | BICF2P457587 | 0 | 38214595 |
| 27 | Chr27_291 | BICF2S23039330 | 0 | 38652550 |
| 27 | Chr27_292 | TIGRP2P355864_rs8918345 | 0 | 41007296 |
| 27 | Chr27_293 | BICF2S22944389 | 0 | 42116405 |
| 27 | Chr27_294 | BICF2G630154270 | 0 | 44554646 |
| 27 | Chr27_295 | BICF2P273972 | 0 | 48133645 |
| 27 | Chr27_296 | BICF2S23436079 | 0 | 48275088 |
| 28 | Chr28_297 | BICF2G630274021 | 0 | 17525682 |
| 28 | Chr28_298 | BICF2G630273285 | 0 | 19229275 |
| 28 | Chr28_299 | BICF2S23111993 | 0 | 20237946 |
| 28 | Chr28_300 | BICF2G630269861 | 0 | 23907523 |
| 28 | Chr28_301 | BICF2P865971 | 0 | 33946217 |
| 28 | Chr28_302 | BICF2P768331 | 0 | 34484729 |
| 29 | Chr29_303 | BICF2P456086 | 0 | 23130206 |
| 29 | Chr29_304 | BICF2G630624619 | 0 | 26052925 |
| 29 | Chr29_305 | BICF2G630624737 | 0 | 26137527 |
| 29 | Chr29_306 | BICF2G630625275 | 0 | 26681687 |
| 29 | Chr29_307 | BICF2G630629818 | 0 | 33565464 |
| 29 | *Chr29_308 | BICF2S2308013 | 0 | 35329594 |
| 29 | Chr29_309 | BICF2G630634181 | 0 | 38701675 |
| 29 | Chr29_310 | BICF2S23718624 | 0 | 40065218 |
| 30 | Chr30_311 | BICF2P401166 | 0 | 16655286 |
| 30 | Chr30_312 | BICF2G630405361 | 0 | 25154318 |
| 30 | Chr30_313 | BICF2S23239883 | 0 | 39494391 |
| 30 | Chr30_314 | BICF2P875416 | 0 | 42356835 |
| 31 | Chr31_315 | BICF2S2319185 | 0 | 17276416 |
| 31 | Chr31_316 | BICF2P859952 | 0 | 22024791 |
| 31 | Chr31_317 | BICF2P773557 | 0 | 25268139 |
| 31 | Chr31_318 | BICF2P765531 | 0 | 25305414 |
| 31 | Chr31_319 | BICF2G630737601 | 0 | 26943337 |
| 31 | Chr31_320 | BICF2P1169459 | 0 | 39868493 |
| 32 | Chr32_321 | BICF2S23345333 | 0 | 3377035 |
| 32 | Chr32_322 | BICF2P685921 | 0 | 3852899 |
| 32 | Chr32_323 | BICF2P1366125 | 0 | 4277175 |
| 32 | Chr32_324 | BICF2P859938 | 0 | 4321102 |
| 32 | Chr32_325 | BICF2G630600059 | 0 | 8609054 |
| 32 | Chr32_326 | BICF2G630599324 | 0 | 9594107 |
| 32 | Chr32_327 | BICF2P653699 | 0 | 37195940 |
| 33 | Chr33_328 | BICF2G630248709 | 0 | 12442543 |
| 33 | Chr33_329 | BICF2S2361501 | 0 | 25913230 |
| 33 | *Chr33_330 | BICF2P1156327 | 0 | 28616373 |
| 33 | Chr33_331 | BICF2P535120 | 0 | 29705608 |
| 33 | *Chr33_332 | BICF2G63076734 | 0 | 30638645 |
| 33 | *Chr33_333 | BICF2G63076383 | 0 | 30857565 |
| 34 | Chr34_334 | BICF2S23354251 | 0 | 15845567 |
| 34 | Chr34_335 | BICF2P195760 | 0 | 25028785 |
| 34 | Chr34_336 | BICF2G630450299 | 0 | 40054707 |
| 35 | Chr35_337 | BICF2S23224909 | 0 | 17378750 |
| 36 | Chr36_338 | BICF2S23661952 | 0 | 24384176 |
| 37 | Chr37_339 | BICF2P1095742 | 0 | 5214992 |
| 37 | Chr37_340 | BICF2S23256095 | 0 | 17677575 |
| 37 | Chr37_341 | BICF2P1359786 | 0 | 20449627 |
| 38 | Chr38_342 | BICF2S23226863 | 0 | 3245897 |
| 38 | Chr38_343 | BICF2P235721 | 0 | 10694002 |
| 38 | Chr38_344 | BICF2P610285 | 0 | 10842634 |
| 38 | *Chr38_345 | BICF2S23047177 | 0 | 11813014 |
| 38 | Chr38_346 | BICF2P566596 | 0 | 12376019 |
| 38 | Chr38_347 | BICF2P851921 | 0 | 15469064 |
| 38 | Chr38_348 | BICF2G63069395 | 0 | 21427038 |
| 39 | *Chr39_349 | BICF2G6307712 | 103,56 | 101748488 |
| 39 | *Chr39_350 | BICF2G6307705 | 103,56 | 101756307 |
| 39 | *Chr39_351 | BICF2G6307691 | 103,56 | 101797505 |
| 39 | *Chr39_352 | BICF2G6307634 | 103,56 | 101973648 |
| 39 | *Chr39_353 | BICF2P456579 | 129,189 | 124291754 |

^1^ MAP-file format and information: http://pngu.mgh.harvard.edu/~purcell/plink/data.shtml#map

Table S3. Summary of major functional genes near single nucleotide polymorphism (SNP) loci identified as outlier loci and/or associated with environmental variables based on a study of 59 wolves in four European population clusters. Results are grouped into immune system & disease, sensory functions, and brain and cognition. Identification of environmental variables is given in Table 1. Full locus identification from the Illumina CanineHD BeadChip microarray is provided in Supplemental Table S2. Function summary is based on references from the NCBI database (http://www.ncbi.nlm.nih.gov/gene).

| **Chr & SNP number^1^** | **BayeScan log10(PO)^2^** | **BayeScan FDR^3^** | **SAM result^4^** | **Fst^5^** | **Gene(s)** | **Function summary** |
| --- | --- | --- | --- | --- | --- | --- |
|  |  |  |  |  |  | **IMMUNE SYSTEM & DISEASE** |
| Chr7_128 | -- | -- | annt, jant, snow**^6^** (AG,GG) | 0.220 | IL10 | Immunoregulation (pleotropic effects) |
| Chr7_133 | 0.633 (NB)  0.959 (BU) | 0.062 (NB)  0.055 (BU) | bioc (AA,AG) | 0.335 | DSC1/DSG1/DSG3 | Autoimmune skin blistering disease (Pemphigus vulgaris) |
| Chr11_158 | 0.623 (NB) | 0.065 (NB) | -- | 0.3375 | IL4/IL5/IL13 | Immune defense, allergy |
| Chr12_159 | 0.588 (NB) | 0.076 (NB) | bioc (AA,AG) | 0.335 | DLA88/DLA-12/DLA-64/DDX39B/TNF/LY6G5C/AGER/DLA-DRA/HLA-DRB1/DLA-DQA1/DLA-DQB1/PSMB8/DLA-DMB | Immune system (major histocompatibility complex – MHC and vicinity) |
| Chr26_272 | 0.796 (NU) | 0.081 (NU) | -- | 0.232 | ULK1 | Crohn’s disease (inflammatory bowel syndrome) |
| Chr26_277 | 0.863 (NB) | 0.045 (NB) | jant (GG), bioc (GC) | 0.265 | PEBP1 | Systemic inflammatory response syndrome (SIRS) |
| Chr26_281 | 1.547 (4P)  0.779 (NU) | 0.012 (4P)  0.089 (NU) | bioc (GG) | 0.350 | ADORA2A | Implicated in inflammatory diseases and neurodegenerative disorders. |
| Chr27_284 | 0.546 (4P)  1.358 (CU) | 0.105 (4P)  0.035 (CU) | bioc (GG) | 0.521 | FGD4 | Defence against Cryptosporidium parvum, a gastrointestinal parasite. Charcot-Marie-Tooth disease of the peripheral nervous system. |
| Chr28_298 | 0.902 (CU) | 0.071 (CU) | bioc (AG,GG) | 0.185 | COL17A1 | Autoimmune skin blistering disease (Bullous pemphigoid). |
| Chr30_313 | 0.677 (NU) | 0.105 (NU) | -- | 0.158 | NEO1 | Immunoglobulin cell surface protein. |
| Chr31_320 | 0.755 (4P) | 0.076 (4P) | -- | 0.200 | AIRE  ITGB2 | Rare autoimmune disease (polyendocrinopathy)  Canine leukocyte adhesion deficiency (CLAD). |
| Chr38_347 | 1.004 (4P) | 0.039 (4P) | bioc (GG) | 0.335 | USH2A | Usher Syndrome (impairment of hearing and vision (retinis pigmentosa)). |
|  |  |  |  |  |  | **SENSORY** |
| Chr3_32 | 1.011 (4P)  0.874 (BU) | 0.037 (4P)  0.065 (BU) | bioc (GA,AA) | 0.326 | PDE6B | Night blindness, rod/cone dysplasia |
| Chr5_100 | 0.860 (CU) | 0.079 (CU) | bioc (AC) | 0.260 | HSF4 | Heat shock response, cataracts |
| Chr6_121 | 1.243 (4P)  2.853 (NB) | 0.024 (4P)  0.001 (NB) | bioc (AA), lat (CC)^6^ | 0.529 | CLDN9 | Hearing organ |
| Chr9_148 | 1.217 (4P) | 0.027 (4P) | jult (AA) | 0.332 | cOR1P2/OR3A2/OR01E11/OR08H10 | Olfactory receptors |
| Chr20_212 | -- | -- | bioc (GA,AA) | 0.128 | ATXN7 | Retinal degeneration |
| Chr20_214 | 1.522 (NB) | 0.013 (NB) | -- | 0.268 | OLFM2  OR08H09/  OR08C06/  OR7G2 | Ocular disorders, glaucoma  Olfactory receptors |
| Chr21_217 | 0.591 (4P)  1.475 (NB)  0.672 (BU) | 0.096 (4P)  0.014 (NB)  0.096 (BU) | -- | 0.297 | OR4B06/ OR10A4/ cOR10A9/  OR10B10/  OR2D3/ OR10F05 | Olfactory receptors |
| Chr21_218 | -- | -- | bioc (AA,AG) | 0.432 | cOR10AB2/cOR5E1P/cOR10A3 | Olfactory receptors |
| Chr25_268 | 0.771 (BC) | 0.069 (BC) | -- | 0.201 | PDE6D | Retinal rod cells. |
| Chr25_269 | 0.771 (BC) | 0.069 (BC) | bioc (AA) | 0.197 | SAG | Oguchi disease (rare form of night blindness) |
| Chr27_286 | 0.784 (4P)  0.821 (BU) | 0.074 (4P)  0.069 (BU) | bioc (AA) | 0.383 | BHLHE41 | Mammalian circadian clock. Skeletal muscle differentiation. |
| Chr27_287 | 0.820 (4P)  2.032 (NB) | 0.071 (4P)  0.006 (NB) | bioc (AA), jant (GG) | 0.564 | CAFA-T2R46/67 | Bitter taste receptors |
| Chr27_287 | 0.820 (4P)  2.032 (NB) | 0.071 (4P)  0.006 (NB) | bioc (AA), jant (GG) | 0.564 | TASR7/ TASR10/TASR 42 | Bitter taste receptors |
| Chr27_287 | 0.820 (4P)  2.032 (NB) | 0.071 (4P)  0.006 (NB) | lat, jant ^6^(GG), bioc (AA) | 0.564 | CSDA | Epithelial cell signaling in response to cellular stress. |
| Chr27_290 | 1.220 (4P)  2.152 (NB) | 0.026 (4P)  0.004 (NB) | bioc (AA), jant (GG) | 0.571 | TAS2R42 | Bitter taste receptors |
| Chr29_309 | 0.555 (NB) | 0.085 (NB) | lat (GG) | 0.360 | CALB1 | Olfaction. |
|  |  |  |  |  |  | **BRAIN AND COGNITION** |
| Chr17_202 | 0.607 (4P)  1.004 (BC) | 0.094 (4P)  0.048 (BC) | -- | 0.204 | CTNNA2 | Excitement seeking, risk taking |
| Chr21_216 | 1.057 (NB) | 0.032 (NB) | lat, jant^6^(AA) | 0.431 | DLG2 | Complex learning, cognitive flexibility, attention |
| Chr21_222 | 0.629 (NU) | 0.111 (NU) | bioc (AG,GG) | 0.231 | TPH1 | Serotonin biosynthesis |
| Chr22_226 | 0.867 (NC) | 0.082 (NC) | bioc (GG), popd (CC) | 0.320 | HTR2A | Serotonin-receptor |
| Chr23_234 | 0.557 (4P)  0.899 (BU) | 0.103 (4P)  0.062 (BU) | bioc (AG,GG) | 0.242 | CLSTN2 | Memory |
| Chr26_280 | 0.813 (NU) | 0.068 (NU) | bioc (AA), jult (GG) | 0.255 | YWHAH | Mental illness |
| Chr26_281 | 1.547 (4P)  0.779 (NU) | 0.012 (4P)  0.089 (NU) | bioc (GG) | 0.352 | COMT | Brain dopamine level regulation, working memory. [Allele function appears to vary across ethnic groups – Chinese vs. Caucasian]. |
| Chr31_317 | 1.062 (BC) | 0.043 (BC) | bioc (GG) | 0.315 | APP | Canine cognitive dysfunction |
| Chr33_331 | 0.533 (NB) | 0.095 (NB) | lat, alt, ecoc^6^(AA) | 0.422 | ADCY5 | Learning |

^1^Full SNP identification given in Supplemental Table S2.

^2^Pairwise comparisons for: B – Balkan-Dinaric; C – Carpathian Mnts.; U – Ukrainian Steppe; N – Northcentral Europe. 4P: across all four clusters.

^3^False discovery rate threshold (q-value).

^4^Environmental variables identified by the Spatial Analysis Method (SAM) as significantly associated with one or more genotypes. SAM incorporates two separate tests; the Wald and the likelihood ratio (G) test (Joost et al. 2007). The variable “bioc” was identified by the Wald-test; all other variables by the G-test. No result was identified in both.

^5^Fst calculated across all 353 loci for all population clusters.

^6^Correlations between (some) variables. See Table S1 with results for all variable combinations.

Table S4. Functional genes where genotype frequencies show spatial patterns between population clusters (e.g., Northcentral against the other three, or Northcentral and Ukrainian Steppe against others). Genes are located within 1Mb of single nucleotide polymorphism (SNP) loci identified as being outlier loci and/or associated with environmental variables based on a study of 59 wolves in four European population clusters. Full locus identification from the Illumina BeadChip is provided in Supplemental Table S2, with SNPs numbered from 1-353. Function summary is based on references from the NCBI database (http://www.ncbi.nlm.nih.gov/gene). N/A means no obvious gene/function was found, genes reported to affect dogs are highlighted in bold font. Five loci marked with (*) were removed during LD-pruning. Loci marked with (+) showed a high frequency in the given cluster(s), whereas loci marked (–) exhibited a low frequency.

| **Chr & SNP number** | | **Genotype** | | **Gene** | **Function summary** |
| --- | --- | --- | --- | --- | --- |
| **NORTHCENTRAL** | | | | | |
| Chr2_19 | | AG-  GG+ | |  | N/A |
| Chr19_209 | | GG+ | |  | N/A |
| Chr21_223 | | AA+  GA- | | NAV2 | Encodes a member of the neuron navigator gene family, which may play a role in cellular growth and migration. May be involved in the process of neuron growth and regeneration. |
| Chr22_ 232 | | AA+  GA- | | MCF2L  F7  F10 | Osteoarthritis  Essential for hemostasis. Defects in this gene can cause coagulopathy.   Coagulation, mutations of this gene result in factor X deficiency, a hemorrhagic condition of variable severity. |
| Chr24_244 | | AG-  GG+ | |  | N/A |
| Chr26_ 272 | | CC+  CG- | | PGAM5 | Drosophila: heat shock response. |
| Chr28_ 299 | | GG- | | SORCS | Strongly expressed in the central nervous system. The three SORCS genes likely have diverse, but partly overlapping functions in the developing and mature central nervous system. |
| Chr28_301 | | AA+  GA- | |  | N/A |
| Chr38_ 347 | | AG+  GG- | | USH2A | Encoded protein may be important in development and homeostasis of the inner ear and retina. Mutations within this gene have been associated with Usher syndrome type IIa and retinitis pigmentosa. Usher Syndrome type II (USH2) is an autosomal recessive disorder, characterized by moderate to severe hearing impairment and retinitis pigmentosa (RP). |
| Chr15_ 185 | | GA+ | | GRIA2 | Glutamate receptors are the predominant excitatory neurotransmitter receptors in the mammalian brain and are activated in a variety of normal neurophysiologic processes. |
| Chr1_9 | | AA+  GA- | |  | N/A |
| Chr9_150 | | AA- | |  | N/A |
| Chr12_163 | | GG+ | |  | N/A |
| Chr25_255* | | CC+ | |  | N/A |
| **NORTHCENTRAL and UKRAINIAN STEPPE** | | | | | |
| Chr2_18 | | AA+ | |  | N/A |
| Chr26_273 | | GG- | |  | N/A |
| Chr1_4 | | AA+ | |  | N/A |
| **CARPATHIAN MOUNTAINS** | | | | | |
| Chr1_10 | | AA+  GG- | |  | N/A |
| Chr2_ 11 | | GG- | | SPAG6  MLLT10  NEBL | Sperm flagellar motility; maintenance of structural integrity of mature sperm.  Leukemia  Cardiac muscle, cardiomyopathy (weakening of heart muscle). |
| Chr6_124 | | AA+ | |  | N/A |
| Chr6_125 | | AA+  GG- | |  | N/A |
| Chr8_142 | | AA+ | |  | N/A |
| Chr10_152 | | GA- | |  | N/A |
| Chr10_157 | | AA-  GG+ | |  | N/A |
| Chr12_161 | | AA+ | |  | N/A |
| Chr13_173 | | AA+  GG- | |  | N/A |
| Chr14_ 178 | | GG+ | | ISPD | Mutations in this gene are the cause of Walker-Warburg syndrome, an inherited disorder that affects development of the muscles, brain, and eyes, and the most severe of a group of genetic conditions known as congenital muscular dystrophies, which cause muscle weakness and wasting (atrophy) beginning very early in life |
| Chr14_ 179 | | GG+  GA- | |  | N/A |
| Chr15_ 184* | | GG- | | LRBA | Defects in this gene are associated with the disorder common variable immunodeficiency-8 with autoimmunity. |
| Chr16_196 | | AA+  GG- | |  | N/A |
| Chr22_ 226 | | CC-  GC+ | | HTR2A | Encodes one of the receptors for serotonin, a neurotransmitter with many roles. Mutations in this gene are associated with susceptibility to schizophrenia and obsessive-compulsive disorder. |
| Chr13_ 175 | | AA+ | | PDGFRA  **KIT**  **KDR (VEGFR2)** | Likely plays a role in organ development, wound healing, and tumor progression.  **Dog coat pattern:** **Spotted Weimaraner dog due to de novo KIT mutation*.***  **Canine ocular disease.** |
| Chr3_ 23 | | GG+ | | **IGF1R** | C**ontributes to reduced size in dogs, affects dogs, cattle, and humans.** |
| Chr3_ 24 | | AA+  AG+  GG- | | ST8SIA2 | Mental illness in humans, promotes fear behavior in mice. |
| Chr25_253 | | AA+ | |  | N/A |
| Chr25_ 269 | | AA+ | | SAG  TRPM8 | S-arrestin (S-antigen) is a major soluble photoreceptor protein involved in desensitization of the photoactivated transduction cascade. Expressed in the retina and the pineal gland and inhibits coupling of rhodopsin to transducin in vitro. Highly antigenic, capable of inducing experimental autoimmune uveoretinitis. Mutations associated with Oguchi disease, a rare autosomal recessive form of night blindness.  TRPM8 belongs to the family of transient receptor potential channels and is activated by cooling and cooling agents, such as icilin and menthol. It is expressed in a subset of sensory neurons and involved in thermosensation. Acts as a cold sensor, having an activation threshold of ∼28°C. TRPM8 neurons are required for the breadth of behavioral responses evoked by cold temperatures. |
| Chr26_ 279 | | AA+ | | CRYBB3  CRYBB2  CRYBB1  CRYBA4 | Vertebrate eye lens, mutations in this gene result in cataract congenital nuclear autosomal recessive type 2.  Vertebrate eye lens.  Vertebrate eye lens.  Vertebrate eye lens. |
| Chr3_27 | | GG+ | |  | N/A |
| Chr27_ 284 | | AA-  GA+  GG+ | | PKP2  FGD4 | Possibly linked to arrhythmogenic right ventricular cardiomyopathy (ARVC), and skin structure.  Plays a role in mediating the cellular invasion of Cryptosporidium parvum, an intracellular parasite that infects the gastrointestinal tract. Mutations in this gene can cause Charcot-Marie-Tooth disease type 4H (CMT4H), a disorder of the peripheral nervous system. CMT results in progressive loss of muscle tissue and touch sensation across various parts of the body. |
| Chr27_ 291 | | AA+  GA- | | KLRK1 | Naturally occurring read-through transcription between the neighboring KLRC4 (killer cell lectin-like receptor subfamily C, member 4) and KLRK1 (killer cell lectin-like receptor subfamily K, member 1) genes on chromosome 12. Possible immune system function. |
| Chr31_ 317 | | GG+ | | ADAMTS5  **APP**  ADAMTS1 | The enzyme encoded by this gene contains two C-terminal TS motifs and functions as aggrecanase to cleave aggrecan, a major proteoglycan of cartilage. Likely related to deformation and destruction of human temporomandibular joint (TMJ) discs.  Amyloid β peptide is generated from the sequential protease cleavage of the amyloid precursor protein (APP). Accumulation of Aβ peptide in the cortical and hippocampal brain regions is a major pathological feature of Alzheimer's disease (AD). **Canine cognitive dysfunction syndrome (CDS, similar to early AD) in aged dogs appears to be accompanied by cortical deposition of Aβ peptides and neurodegeneration.**  This gene is likely to be necessary for normal growth, fertility, and organ morphology and function. |
| Chr31_ 320 | | AA- | | TMPRSS3  HSF2BP  AIRE  **ITGB2** | Association with both congenital and childhood onset autosomal recessive deafness, thought to be involved in the development and maintenance of the inner ear.  Heat shock transcription factor 2 binding protein.   Important role in immunity by regulating the expression of autoantigens and negative selection of autoreactive T-cells in the thymus. Mutations in this gene cause the rare autosomal-recessive systemic autoimmune disease termed autoimmune polyendocrinopathy with candidiasis and ectodermal dystrophy (APECED).  **Immune response (dog, human), defects in this gene cause leukocyte adhesion deficiency. Canine leukocyte adhesion deficiency (CLAD).** |
| Chr38_ 342 | | AA-  GA+ | | REN | Blood pressure and electrolyte balance. |
| Chr5_ 100 | | AA+  CC- | | TK2  **HSF4** | The encoded enzyme localizes to the mitochondria and is required for mitochondrial DNA synthesis. Mutations in this gene are associated with a myopathic form of mitochondrial DNA depletion syndrome.  Heat-shock transcription factors (HSFs) activate heat-shock response genes under conditions of heat or other stresses. **Associated with the development of cataracts (leading cause of blindness) in dogs.** |
| **CARPATHIAN MOUNTAINS and UKRAINIAN STEPPE** | | | | | |
| Chr2_ 13 | AA-  GA+ | | CUBN  **PTPLA**  VIM | | Cubilin (CUBN) acts as a receptor for intrinsic factor-vitamin B12 complexes. The role of receptor is supported by the presence of 27 CUB domains. Cubulin is located within the epithelium of intestine and kidney. Mutations in CUBN may play a role in autosomal recessive megaloblastic anemia.  PTP family are signaling molecules that regulate a variety of cellular processes. This gene was preferentially expressed in both the adult and fetal heart. The tissue specific expression in the developing and adult heart suggests a role in regulating cardiac development and differentiation. **Centronuclear myopathies (CNM, inherited congenital disorders characterized by an excessive number of internalized nuclei) in Labradors**.  The protein encoded is responsible for maintaining cell shape, cytoplasm integrity, and stabilizing cytoskeletal interactions. Involved in immune response; controls transport of low-density lipoprotein (LDL)-derived cholesterol from a lysosome to the site of esterification. Likely helps regulate smooth muscle cells. Vimentin filaments may play an important role in mediating active force development and passive tension. |
| SAM 163 | AG+ | |  | | N/A |
| SAM 270 | AA- | |  | | N/A |
| **CARPATHIAN MOUNTAINS and DINARIC-BALKAN** | | | | | |
| Chr10_156 | AA+ | | REV1 | | Encodes Rev1 proteins that contain a BRCT domain important in protein-protein interactions. Human Rev1-like protein may be a scaffold that recruits DNA polymerases involved in translesion synthesis (TLS) of damaged DNA. |
| Chr15_ 185 | AA+ | | GRIA2 | | Glutamate receptors are the predominant excitatory neurotransmitter receptors in the mammalian brain and are activated in a variety of normal neurophysiologic processes. |
| Chr6_120 | AA-  GG+ | |  | | N/A |
| Chr6_126 | AA+  GG- | |  | | N/A |
| Chr11_158 | AA- | | **IL5**  **IL13**  **IL4**  **GDF-9**  HSPA4 | | **Dogs: allergies.**    **Dogs, human, mice: immune defense*.***  **Dogs, humans: immune defense.**  **Canine GDF-9 could be causatively linked to the unique ovulation process in the Canidae.**  Heat shock proteins (HSPs) are proteins that are expressed under variety of stresses including pathologic conditions. |
| Chr16_195 | AG+  GG- | | TUSC3 | | Candidate tumor suppressor gene, expressed in most nonlymphoid human tissues including prostate, lung, liver, and colon. Defects in the TUSC3 gene identified in individuals with nonsyndromic autosomal recessive intellectual disability (ARID). |
| Chr21_217 | AA- | | LOC485347  LOC485348  LOC100688800  OR4B06  OR10A4  LOC100688871  cOR10A9  OR10B10  OR2D3  LOC485358  LOC485359  LOC100682782  OR10F05 | | Olfactory receptors  Olfactory receptor  Olfactory receptor  Olfactory receptor  Olfactory receptor  Olfactory receptor  Olfactory receptor  Olfactory receptor  Olfactory receptor  Olfactory receptor  Olfactory receptor  Olfactory receptor  Olfactory receptor |
| Chr31_318 | AG-  GG+ | |  | | N/A |
| Chr33_331 | AA-  CA+ | | ADCY5 | | Humans: SNPs in this gene may be associated with low birth weight and type 2 diabetes. Mice: AC5 may be specifically required for learning associations between discrete cues and outcomes in which the temporal relationship between conditioned stimulus (CS) and unconditioned stimulus (US) is essential. In addition, loss of AC5 compromises the ability of both contextual and discrete cues to modulate instrumental behavior. |
| Chr37_ 341 | AA+  GA- | | **MAP2** | | **Immunoreactivity in the lumbar spinal cord of young adult and aged dogs.** |
| Chr38_343 | AA+ | |  | | N/A |
| Chr4_ 46 | AA- | | ZFR | | Zinc finger RNA binding protein, RNA regulation |
| Chr21_215 | GG+ | | FZD4  TMEM126A | | Familial exudative vitreoretinopathy (FEVR), causing vision loss   Defects in this gene are a cause of optic atrophy type 7 (OPA7) |
| Chr24_245 | CC+ | | BMP7 | | The bone morphogenetic proteins (BMPs) are a family of secreted signaling molecules that can induce ectopic bone growth, and promote osteoblast differentiation and bone mineralization. The BMP encoded by this gene expressed early embryogenesis; proposed role in early development and possible bone inductive activity. |
| Chr35_337 | GG+ | |  | | N/A |
| **UKRAINIAN STEPPE** | | | | | |
| Chr7_131 | AA+ | |  | | N/A |
| Chr7_136 | AA- | |  | | N/A |
| Chr7_137 | AA+  GG- | |  | | N/A |
| Chr9_ 143 | AA+ | | RPTOR | | Encodes a component of a signaling pathway that regulates cell growth in response to nutrient and insulin levels. Moyamoya disease (brain arteries constricted). Climate adaptation; derived allele may influence thermogenesis or immune response by altering MTOR pathway activity and thereby increasing fitness in colder climates. |
| Chr9_144 | GG- | |  | | N/A |
| Chr9_145 | CC- | |  | | N/A |
| Chr10_152 | GG+ | |  | | N/A |
| Chr12_162 | AG+  GG- | |  | | N/A |
| Chr13_172 | AG+  GG- | |  | | N/A |
| Chr15_ 187 | GA- | | **FNIP2** | | **Mutation in Weimaraner dogs resulted in hypomyelination of the brain (tremors) and a tract-specific myelin defect in the spinal cord.** |
| Chr2_18 | GG- | |  | | N/A |
| Chr31_318 | AA+ | |  | | N/A |
| Chr13_171 | AG+ | |  | | N/A |
| Chr13_ 169 | GG+ | | ZFPM2  ABRA (STARS)  **RSPO2** | | Tetralogy of Fallot; a congenital heart defect  Actin-binding protein specifically expressed in cardiac, skeletal and smooth muscle; upregulated in response to acute endurance exercise.  **Dog coat color** |
| Chr16_ 194 | AC+  CC- | | SGCZ | | The sarcoglycan complex is part of the dystrophin-associated glycoprotein complex (DGC), which bridges the inner cytoskeleton and the extra-cellular matrix. CNVs that overlap the PARK2, GYPA, and SGCZ genes found associated with BMI, obesity, and other obesity-related traits. |
| Chr1_ 1 | AG+  GG- | | TMX3 | | Eye development; small-eye phenotype |
| Chr17_ 201 | AA+ | | POMC | | This gene encodes a polypeptide hormone precursor that undergoes extensive, tissue-specific, post-translational processing. Mutations in this gene have been associated with early onset obesity, adrenal insufficiency, and red hair pigmentation. |
| Chr17_203 | AA+ | |  | | N/A |
| Chr17_ 205 | GG+ | | VTCN1 | | Known to play a pivotal role in regulation of the immune system and, in soluble form, has previously been associated with higher disease activity |
| Chr18_ 207 | GG- | | EXT2  ACCS (ACS)  PNPLA2  TNNI2  **TH**  KCNQ1 | | Mutations in this gene cause the type II form of multiple exostoses, defined as benign growths of bone extending outwards from the surface of a bone.  Plants: associated with the response to low light intensities.  Hydrolysis of triglycerides in adipose tissue; mutations are associated with neutral lipid storage disease with myopathy.  Regulation of striated muscle contraction, also present in vascular smooth muscle and may play a role in regulation of smooth muscle function. Mutations cause myopathy and distal arthrogryposis type 2B  **Dog behavior: activity, impulsivity and inattention**  Cardiac function, mutations in this gene are associated with hereditary long QT syndrome 1 (also known as Romano-Ward syndrome), Jervell and Lange-Nielsen syndrome, and familial atrial fibrillation. |
| Chr3_ 20 | AC+  CC- | | WDR36  **TSLP** | | Humans; mutations associated with adult-onset glaucoma. Mice; retinal degeneration, mutation to this gene can cause devastating retinal damage.  **Pathogenesis of canine atopic dermatitis (CAD) similar to that in humans (AD)**. Orthologs in mice, humans. The protein promotes T helper type 2 (TH2) cell responses that are associated with immunity in various inflammatory diseases, including asthma, allergic inflammation and Chronic obstructive pulmonary disease. |
| Chr21_219 | AG+ | |  | | N/A |
| Chr3_21 | AG+  GG- | | MAN2A1 | | Humans, mice: mutations in the mouse homolog of this gene have been shown to cause a systemic autoimmune disease similar to human systemic lupus erythematosus. |
| Chr21_220 | AG+  GG- | |  | | N/A |
| Chr22_231 | CC-  GC+ | | COL4A1 | | Mutations in this gene cause porencephaly, cerebrovascular disease, and renal and muscular defects. |
| Chr23_233 | AA+ | |  | | N/A |
| Chr23_236 | AG+ | | WWTR1  AGTR1  HPS3  CP | | May be involved in regulation of extracellular matrix, cell adhesion sites, cell shape and the actomyosin cytoskeleton in vertebrates. Biologically potent transcriptional coactivator; downstream element of the Wnt/β-catenin cascade. May play a role in the onset and progression of glaucoma.  Angiotensin II is important in controlling blood pressure and volume in the cardiovascular system. This gene encodes the type 1 receptor which is thought to mediate the major cardiovascular effects of angiotensin II.  Mutations in this gene are associated with Hermansky-Pudlak syndrome type 3, characterized by oculocutaneous albinism and prolonged bleeding.  Encodes metalloprotein involved in the peroxidation of Fe(II)transferrin to Fe(III) transferrin. Mutations cause aceruloplasminemia, which results in iron accumulation and tissue damage, and is associated with diabetes and neurologic abnormalities. |
| Chr24_239 | AG+  GG- | |  | | N/A |
| Chr24_ 240 | AG+  GG- | |  | | N/A |
| Chr24_ 243 | AG+  GG- | | BCL2L1 | | The proteins encoded by this gene are located at the outer mitochondrial membrane, and regulate outer mitochondrial membrane channel (VDAC) opening. VDAC regulates mitochondrial membrane potential, and controls the production of reactive oxygen species and release of cytochrome C by mitochondria, both of which are the potent inducers of cell apoptosis (programmed cell death). |
| Chr25_271 | AA-  CA+ | |  | | N/A |
| Chr26_ 273 | AA+ | | MMP17 | | Proteins of the matrix metalloproteinase (MMP) family are involved in the breakdown of extracellular matrix in normal physiological processes, such as embryonic development, reproduction, and tissue remodelling, as well as in disease processes, such as arthritis and metastasis. |
| Chr26_278 | CC- | |  | | N/A |
| Chr3_27 | AA+ | |  | | N/A |
| Chr26_281 | AA-  GA+ | | ADORA2A  COMT | | Protein plays an important role in many biological functions, e.g. cardiac rhythm and circulation, cerebral and renal blood flow, immune function, pain regulation, and sleep. Implicated in pathophysiological conditions such as inflammatory diseases and neurodegenerative disorders.  COMT plays a crucial role in regulating brain dopamine level. Associated with working memory (WM) and hippocampus size of the brain. Allele function appears to vary across different ethnic groups, with Chinese subjects showing an opposite pattern as that for Caucasians. |
| Chr27_289 | AA- | |  | | N/A |
| Chr28_302 | AA-  CC+ | |  | | N/A |
| Chr29_304 | GA- | |  | | N/A |
| Chr29_305 | AA- | |  | | N/A |
| Chr31­_315 | AG+  GG- | |  | | N/A |
| Chr3_ 31 | AC+  CC- | | KCNIP4 | | Humans: likely plays a role in ADHD and personality disorders |
| Chr32_321 | AG+  GG- | | LOC100688595 | | Uncharacterized locus. |
| Chr32_327 | AG+  GG- | |  | | N/A |
| Chr33_328 | AG+  GG- | |  | | N/A |
| Chr3_ 32 | AA-  GG+ | | **PDE6B** | | Mutations in this gene result in retinitis pigmentosa and autosomal dominant congenital stationary night blindness. **A mutation (insertion) co-segregates with disease status (generalized progressive retinal atrophy (gPRA)) in a large pedigree of Sloughi dogs. Rod/cone dysplasia 1 in Irish setters.** |
| Chr35_337 | AA+ | |  | | N/A |
| Chr37_339 | GG- | |  | | N/A |
| Chr37_340 | AT+  TT- | |  | | N/A |
| Chr4_ 35 | AA-  GA+ | | KCNMA1  DLG5  RPS24 | | Control of smooth muscle tone and neuronal excitability.   Ulcerative colitis   Humans: mutations result in Diamond-Blackfan anemia. |
| Chr1_ 8 | AG+  GG- | | KCTD15  CHST8  PEPD  CEP89  **SLC7A9**  RGS9BP | | Humans: obesity  Humans: autosomal recessive peeling skin syndrome.  Humans: mutations result in prolidase deficiency (collagen metabolism - connective tissues).  Humans, Drosophila: mitochondrial metabolism, required for neuronal and cognitive function across evolution.  **Dogs, humans: cystinuria, genetic disorder in the domestic dog that leads to recurrent urolith formation**.  Mice, cattle: retina function, mutations associated with prolonged electroretinal response suppression (PERRS), aka. bradyopsia. |
| Chr5_94 | CC- | |  | | N/A |
| Chr5_95* | AA+ | |  | | N/A |
| Chr31_ 320 | AA+ | | TMPRSS3  HSF2BP  AIRE  **ITGB2** | | Association with both congenital and childhood onset autosomal recessive deafness, thought to be involved in the development and maintenance of the inner ear.  Heat shock transcription factor 2 binding protein  Important role in immunity by regulating the expression of autoantigens and negative selection of autoreactive T-cells in the thymus. Mutations in this gene cause the rare autosomal-recessive systemic autoimmune disease termed autoimmune polyendocrinopathy with candidiasis and ectodermal dystrophy (APECED).  **Immune response (dog, human), defects in this gene cause leukocyte adhesion deficiency. Canine leukocyte adhesion deficiency (CLAD).** |
| Chr17_ 205 | AA- | | VTCN1 | | Pivotal role in regulation of the immune system and has been associated with higher disease activity. |
| **UKRAINIAN STEPPE and DINARIC-BALKAN** | | | | | |
| Chr7_ 128 | AG+ | | ASPM | | Participates in spindle organisation, spindle positioning and cytokinesis. Ortholog of the Drosophila 'abnormal spindle' gene (asp), essential for normal mitotic spindle function in embryonic neuroblasts. Studies in mice suggest a role in mitotic spindle regulation, with a preferential role in regulating neurogenesis. Mutations are associated with microcephaly primary type 5 (neurodevelopmental disorder, small head). |
| Chr7_135 | AG+ | |  | | N/A |
| Chr12_ 167 | AA+  CC- | | ZBTB24  FIG4 | | Humans: mutations cause immunodeficiency-centromeric instability-facial anomalies syndrome-2 skeletal muscle function.  Mutations have been associated with Charcot-Marie-Tooth disease, type 4J. |
| Chr13_ 169 | GG+ | | ZFPM2  ABRA (STARS)  **RSPO2** | | Tetralogy of Fallot, a congenital heart defect.  Actin-binding protein specifically expressed in cardiac, skeletal and smooth muscle; up-regulated in response to acute endurance exercise.  **Dog coat color** |
| Chr13_170 | AA-  GA+ | |  | | N/A |
| Chr13_171 | GG- | |  | | N/A |
| Chr13_173 | AG- | |  | | N/A |
| Chr13_ 175 | AG- | | PDGFRA  **KIT**  **KDR (VEGFR2)** | | Likely plays a role in organ development, wound healing, and tumor progression.  **Dog coat pattern: Spotted Weimaraner dog due to de novo KIT mutation*.***  **Canine ocular disease** |
| Chr2_17 | AA- | | YIPF5 | | Mammals: structures endoplasmic reticulum, connection with Golgi apparatus. |
| Chr26_ 280 | GG- | | **SLC5A1**  YWHAH | | The encoded integral membrane protein is the primary mediator of dietary glucose and galactose uptake from the intestinal lumen. Mutations in this gene have been associated with glucose-galactose malabsorption. **Dog (vs. cat) carbohydrate digestion and absorption; dogs have a digestive ability to cope with diets containing significant levels of carbohydrate.**  Association of variants in YWHAH with major mental illness; early-onset schizophrenia and psychotic bipolar disorder. May be associated with osteoarthritis caused by cranial cruciate ligament rupture (CCLR). |
| Chr27_ 291 | GG+ | | KLRK1 | | Naturally occurring read-through transcription between the neighboring KLRC4 (killer cell lectin-like receptor subfamily C, member 4) and KLRK1 (killer cell lectin-like receptor subfamily K, member 1) genes on chromosome 12. Possible immune system function. |
| Chr28_ 297 | AA- | | KAZALD1 | | Mice: possible function in bone development and bone regeneration. |
| Chr29_306 | AA-  GG+ | |  | | N/A |
| Chr29_307 | AA+ | |  | | N/A |
| Chr29_ 309 | GG- | | **CALB1** | | **CB-immunoreactive neurons are involved in the main olfactory bulb of the dog, appears to be reduced with ageing.** |
| Chr29_310 | GG- | |  | | N/A |
| Chr31_319 | AA-  GA+ | |  | | N/A |
| Chr32_324 | AG+  GG-  AA- | |  | | N/A |
| Chr34_ 334 | AA- | | GNB4 | | Heterotrimeric guanine nucleotide-binding proteins (G proteins), which integrate signals between receptors and effector proteins, are composed of an alpha, a beta, and a gamma subunit. This gene encodes a beta subunit; important regulators of alpha subunits and certain signal transduction receptors and effectors. GNB4 mutations are cause of dominant intermediate Charcot-Marie-Tooth disease (CMT), a heterogeneous group of inherited neuropathies. |
| Chr36_ 338 | GG- | | PDE11A | | This gene encodes a member of the PDE protein superfamily. Mutations in this gene are a cause of Cushing disease (prolonged exposure to inappropriately high levels of cortisol) and adrenocortical hyperplasia (increase in the number of cells). |
| Chr4_ 34 | AA+ | | ANK3 | | Family of proteins that are believed to link the integral membrane proteins to the underlying spectrin-actin cytoskeleton and play key roles in activities such as cell motility, activation, proliferation, contact, and the maintenance of specialized membrane domains. Bipolar disorder, schizophrenia. |
| Chr4_ 43 | AA+ | | **SGCD**  HAND1 | | Mutations associated with autosomal recessive limb-girdle muscular dystrophy and dilated cardiomyopathy:  Essential role in cardiac morphogenesis, function in the formation of the right ventricle and aortic arch arteries, implicating them as mediators of congenital heart disease. |
| **DINARIC-BALKAN** | | | | | |
| Chr6_ 113 | AG-  GG+ | | LMTK2 | | Belongs to protein kinase superfamily and the protein tyrosine kinase family. Involved in nerve growth factor (NGF)-TrkA signaling; critical role in endosomal membrane trafficking. Mouse studies suggest essential role in spermatogenesis. |
| Chr29_ 309 | AG+ | | **CALB1** | | **CB-immunoreactive neurons are involved in the main olfactory bulb of the dog, appears to be reduced with ageing.** |
| Chr11_158 | GG+ | | **IL5**  **IL13**  **IL4**  **GDF-9**  HSPA4 | | **Dogs: allergies.**    **Dogs, human, mice: immune defense*.***  **Dogs, humans: immune defense**  **Canine GDF-9 could be causatively linked to the unique ovulation process in the Canidae.**  Heat shock proteins (HSPs) are proteins that are expressed under variety of stresses including pathologic conditions. |
| Chr6_ 121 | AA+  AC+  CC- | | DNASE1  CLDN9 | | Mutations in this gene have been associated with systemic lupus erythematosus (SLE), an autoimmune disease. Reduction in renal Dnase1 expression and activity may be a critical event in the development of severe forms of lupus nephritis.  Claudin-9 may be required for the preservation of sensory cells in the hearing organ. CLDN6 and CLDN9 were able to mediate the entry of HCV (Hepatitis C virus) into target cells. |
| Chr6_123 | AA+  GG- | |  | | N/A |
| Chr7_ 128 | GG- | | ASPM  IL10 | | Participates in spindle organisation, spindle positioning and cytokinesis. Ortholog of the Drosophila 'abnormal spindle' gene (asp), essential for normal mitotic spindle function in embryonic neuroblasts. Mouse studies suggest a role in mitotic spindle regulation, with a preferential role in regulating neurogenesis. Mutations are associated with microcephaly primary type 5 (neurodevelopmental disorder, small head).  Possible role for IL-6, IL-8 and IL-10 in canine mammary malignancy and specific differences in interleukine (Ils) content in Inflammatory mammary cancer (IMC) versus non-IMC MMT that could have future diagnostic and therapeutic implications. Pleiotropic effects in immunoregulation and inflammation. Likely essential immunoregulator in the intestinal tract. |
| Chr7_129 | TT+ | |  | | N/A |
| Chr7_132 | CC-  GC+ | |  | | N/A |
| Chr7_133 | AA+ | | DSG3  DSG1  DSC1  DSC2 | | The encoded protein identified as autoantigen of the autoimmune skin blistering disease pemphigus vulgaris.  The encoded protein identified as autoantigen of the autoimmune skin blistering disease pemphigus foliaceus.  DSC1 may be a major autoantigen in canine Pemphigus foliaceus (PF).  Desmosomal family members are found primarily in epithelial cells where they constitute the adhesive proteins of the desmosome cell-cell junction and are required for cell adhesion and desmosome formation. Mutations in this gene are associated with arrhythmogenic right ventricular dysplasia-11. Desmocollin 2 is a key membrane glycoprotein critically involved in cell-cell adhesion and the maintenance of normal tissue architectures in epithelia. |
| Chr9_149 | AA-  GG+ | | RAP1GAP2 | | Encodes a GTPase-activating protein that activates the small guanine-nucleotide-binding protein Rap1 in platelets. The protein regulates secretion of dense granules from platelets at sites of endothelial damage. |
| Chr9_151 | CC- | | PTGES  CRAT  **DNM1** | | Possible influence of PTGES polymorphism on the pathogenesis of rheumatoid arthritis (RA) and on disease severity through upregulation of mPGES-1 at the sites of inflammation.  Carnitine acetyltransferase (CRAT) is an important enzyme for energy homeostasis and fat metabolism.  **A canine DNM1 mutation is highly associated with the syndrome of exercise-induced collapse**. |
| Chr10_154* | AG+  GG- | |  | | N/A |
| Chr13_176 | AA- | |  | | N/A |
| Chr15_ 183 | AA+ | | **ATP2B1** | | Enzymes remove bivalent calcium ions from eukaryotic cells against very large concentration gradients and play a critical role in intracellular calcium homeostasis. Humans: arterial stiffness and hypertension. **Dogs: vascular smooth muscle cells, possibly Chagas disease (American trypanosomiasis).** |
| Chr10_186* | AA+ | |  | | N/A |
| Chr15_ 188 | GG+ | | **NPY1R** | | **NPY Y(1) receptors appears to produce vasoconstriction in exercising skeletal muscle of dogs.** |
| Chr16_193 | GG+ | |  | | N/A |
| Chr17_197 | AA-  GG+ | |  | | N/A |
| Chr17_198 | AA+  AG+  GG- | |  | | N/A |
| Chr17_206 | CC+  CG+  GG- | |  | | N/A |
| Chr19_211 | GG+ | |  | | N/A |
| Chr20_213 | AC+  CC- | |  | | N/A |
| Chr20_214 | AA+ | | OLFM2  LOC484973 (OR7D4)  **OR08H09**  **OR08C06**  **OR7G2** | | Ocular disorders, glaucoma.  Olfactory receptor (the olfactory receptor gene family is the largest in the genome).  **Dogs: olfactory receptor.**  **Dogs: olfactory receptor*.***  **Olfactory receptor.** |
| Chr21_216 | TT+  AA- | | DLG2 | | Protein forms heterodimer with a related family member that may interact at postsynaptic sites to form a multimeric scaffold for the clustering of receptors, ion channels, and associated signaling proteins. DLG2 role in complex learning, cognitive flexibility and attention has been highly conserved over 100 million years. DLG-family mutations underlie psychiatric disorders, suggesting that genome evolution expanded the complexity of vertebrate cognition at the cost of susceptibility to mental illness. |
| Chr21_217 | CC+ | | PPFIBP2 | | The encoded protein is a beta liprin and plays a role in axon guidance and neuronal synapse development. |
| Chr21_218 | AG+  GG- | | cOR10AB2  cOR5E1P  cOR10A3 | | Olfactory receptor  Olfactory receptor  Olfactory receptor |
| Chr21_219 | AA+ | |  | | N/A |
| Chr3_22 | GG+ | |  | | N/A |
| Chr24_ 242 | AG+  GG- | | C20orf27  PRND  PRNP  MAVS  AVP | | No obvious function reported.  Prion protein. The encoded protein is a membrane glycosylphosphatidylinositol-anchored glycoprotein that is found predominantly in testis. Mutations in this gene may lead to neurological disorders. Associated with Alzheimer's disease (AD).  Prion protein. Mutations in the repeat region as well as elsewhere in this gene have been associated with Creutzfeldt-Jakob disease, fatal familial insomnia, Gerstmann-Straussler disease, Huntington disease-like 1, and kuru.  Encodes an intermediary protein necessary in the virus-triggered beta interferon signaling pathways. It is required for activation of transcription factors which regulate expression of beta interferon and contributes to antiviral immunity. The comprehensive regulation of MAVS in response to foreign RNA may be essential to antiviral host defenses.  Has a direct antidiuretic action on the kidney, and causes vasoconstriction of the peripheral vessels. This hormone can contract smooth muscle during parturition and lactation. It is also involved in cognition, tolerance, adaptation and complex sexual and maternal behaviour, as well as in the regulation of water excretion and cardiovascular functions. |
| Chr24_ 246 | GG+ | | SLC17A9  **COL9A3** | | SLC17A9 protein is a vesicular nucleotide transporter and should lead to the elucidation of the molecular mechanism of ATP secretion in purinergic signal transmission. Likely involvement in ATP release and T cell activation.  Encodes 1 of the 3 alpha chains of type IX collagen, the major collagen component of hyaline cartilage and usually found in tissues containing type II collagen, a fibrillar collagen. Mutations in this gene are associated with multiple epiphyseal dysplasia type 3. **Affected dogs exhibit short-limbed dwarfism and severe ocular defects.** |
| Chr25_ 252 | AG+  GG- | | CLCN3 | | This protein plays a role in both acidification and transmitter loading of GABAergic synaptic vesicles, and in smooth muscle cell activation and neointima formation. ClC-3 is a key component of native volume-sensitive outwardly rectifying anion channels (VSOACs, play a significant role in cell volume homeostasis in mammalian cells) in mammalian heart and plays a significant cardioprotective role against cardiac hypertrophy and failure. |
| Chr26_275 | AC+  CC- | |  | | N/A |
| Chr26_277 | CC-  GG+ | | CCDC60  PEBP1 | | No obvious function reported.  Raf kinase inhibitor protein (RKIP) regulates MAPK and NF-κB signaling cascades. Both critical for production of proinflammatory cytokines responsible for initiation of systemic inflammatory response syndrome (SIRS) associated with development of severe medical complications, including progression to multiple organ dysfunction syndrome and death. |
| Chr27_286 | AA+  TT- | | BHLHE41 | | The encoded protein is a key regulator of the mammalian circadian clock. A dominant mutation in this gene results in a short sleep phenotype. May influence (inhibit) skeletal muscle differentiation. |
| Chr27_287 | AA+  AG+  GG- | | CAFA-T2R67  CAFA-T2R43  CSDA | | Bitter taste receptor (T2R) gene (15 in dogs, 25 in humans, 34 in mice etc).  Bitter taste receptor. Bitter taste is a basic taste modality, required to safeguard animals against consuming toxic substances. The human TAS2R10 responds to the toxic strychnine and numerous other compounds. Possibly independent evolution of various strychnine-binding sites.  May be associated with signalling in epithelial cells in response to cellular stress. |
| Chr27_288 | AA+  AG+  GG- | |  | | N/A |
| Chr27_ 290 | AA+  AG+  GG- | | TAS2R42 | | Taste receptor. Many natural poisonous substances are bitter, and the mammalian T2R genes may be under diversifying selection for ability to recognize an array of poisons the organisms may encounter in exploring new habitats and diets. |
| Chr27_295 | AA+ | |  | | N/A |
| Chr27_296 | AA-  GG+ | | ATP6V1E1  CACNA1C | | Encodes a component of V-ATPase, an enzyme that mediates acidification of eukaryotic intracellular organelles. Acidification is necessary for such intracellular processes as protein sorting, zymogen activation, receptor-mediated endocytosis, and synaptic vesicle proton gradient generation. Amplification of genes CECR2, SLC25A18 and ATP6V1E1, mapping within the critical region for Cat Eye Syndrome (CES), may be responsible for anorectal, renal and preauricular anomalies in patients with CES.  May be implicated in bipolar disorder (BD) and schizophrenia (SZ). Also with cardiac problems (long QT syndrome; rare inherited heart condition with irregular heartbeat). |
| Chr1_ 2 | AA+ | | RAX | | Eye development, retina. |
| Chr29_304 | GG+ | |  | | N/A |
| Chr30_311 | GG- | |  | | N/A |
| Chr31_316 | AA+ | |  | | N/A |
| Chr36_ 338 | AA+ | | PDE11A | | This gene encodes a member of the PDE protein superfamily. Mutations in this gene are a cause of Cushing disease (prolonged exposure to inappropriately high levels of cortisol) and adrenocortical hyperplasia (increase in number of cells). |
| Chr4_ 38 | AG+  GG- | | SLIT3  PANK3  KIBRA (WWC1) | | Major depressive disorder (MDD) and rheumatoid arthritis  Neurodegeneration  Spatial memory |
| Chr4_ 45 | AA-  CC+ | | FYB  LIFR | | Adapter for the FYN protein and LCP2 signaling cascades in T-cells. The encoded protein is involved in platelet activation and controls the expression of interleukin-2.  Leukemia inhibitory factor (LIF) is a pleiotropic cytokine of the IL-6 family that activates the hypothalamic-pituitary-adrenal axis and promotes corticotrope cell differentiation during development. Humans: mutations cause Schwartz-Jampel syndrome type 2, a bent-bone dysplasia disease, and LIFR gene may be related to schizophrenia. |
| Chr4_ 46 | CC+ | | ZFR | | Zinc finger RNA binding protein, RNA regulation |
| Chr1_ 7 | AG+  GG- | | EGLN2  MIA  ITPKT  LTBP4  PRX  PLD3  DYRK1B  DLL3  GMFG  SARS2  SIRT2  **RYR1**  RASGRP4  KCNK6  PPP1R14A (CPI17; CPI-17; PPP1INL) | | Humans: oxygen homeostasis  Humans: melanoma inhibitory activity  Humans: inflammation of blood vessels  Humans: Defects in this gene may be a cause of cutis laxa and severe pulmonary, gastrointestinal, and urinary abnormalities.  Humans: Charcot-Marie-Tooth disease (CMT): one of the most common inherited neurological disorders.  Humans: mutations associated with Alzheimer's risk.  Humans: mutations in this gene were found to cause abdominal obesity-metabolic syndrome 3.  Humans: mutations cause autosomal recessive (AR) spondylocostal dysostoses (SCD) a heterogeneous group of disorders of axial skeletal malformation characterized by multiple vertebral segmentation defects and rib anomalies.  Humans: regulates actin dynamics and contraction in human airway smooth muscle.  Humans: within the critical interval for the autosomal dominant deafness locus DFNA4 and might be linked to this disorder.  Humans: presence of the SIRT2 SNP with APOE ε4-negative status contributes to the development of Alzheimer's disease.  **Dogs, humans: inherited disorders affecting skeletal muscles.**  Humans: possibly affecting inflammatory disorders such as arthritis or inflammatory bowel disease.  Humans; sickle-cell anemia and positional candidate gene for DFNA4, a form of autosomal dominant nonsyndromic hereditary hearing loss.  Humans: encoded protein is inhibitor of smooth muscle myosin phosphatase. |
| Chr17_204 | GG-  AA+ | | AMPD1  CASQ2 | | Skeletal muscle, in humans likely implicated in metabolic myopathy.  Cardiac and slow skeletal muscle cells. Mutations cause stress-induced polymorphic ventricular tachycardia, also referred to as CPVT2, a disease characterized by bidirectional ventricular tachycardia that may lead to cardiac arrest, **canine heart failure**. |
| Chr8_141 | GG+ | |  | | N/A |
| Chr5_ 98 | GG+ | | JUN | | AP-1 and SAF-1 DNA-binding activities are increased in cytokine-stimulated cells as well as in osteoarthritic cartilage tissues. AP-1 signaling pathway may contribute to the physiological impact of the gut microbiota on the host. |

Table S5. Functional genes without obvious spatial patterns. Genes are located within 1Mb of single nucleotide polymorphism (SNP) loci identified as being outlier loci and/or associated with environmental variables based on a study of 59 wolves in four European population clusters. Identification of environmental variables is given in Table 1. Full locus identification from the Illumina BeadChip is provided in Supplemental Table S2, with SNPs numbered from 1-353. Function summary is based on references from the NCBI database (http://www.ncbi.nlm.nih.gov/gene).

| **Chr & SNP number** | **Gene** | **Function summary** |
| --- | --- | --- |
|  |  | **TEMPERATURE** |
| Chr9_148 | TRPV1/TRPV3 | Thermoregulation |
|  |  | **METABOLISM** |
| Chr5_85 | SGIP1 | Fat mass, food intake, fat metabolism |
| Chr18_208 | CPT1A  FGF4 | mtDNA membrane, lipid metabolism  Bone morphogenesis |
| Chr32_326 | SCD5 | Energy metabolism |
|  |  | **PHYSICAL DEVELOPMENT** |
| Chr19_210 | DARS | Hypomyelination (defective myelin formation in the spinal cord and brain) |
| Chr21_222 | HPS5 | Hermansky-Pudlak syndrome (oculocutaneous albinism, platelet abnormality) |
|  |  | **IMMUNE SYSTEM & DISEASE** |
| Chr12_159 | DLA88/DLA-12/DLA-64/DDX39B/TNF/LY6G5C/AGER/DLA-DRA/HLA-DRB1/DLA-DQA1/DLA-DQB1/PSMB8/DLA-DMB | Immune system (major histocompatibility complex – MHC and vicinity) |
| Chr28_298 | COL17A1 | Autoimmune skin blistering disease (Bullous pemphigoid) |
| Chr30_313 | NEO1 | Immunoglobulin cell surface protein |
|  |  | **SENSORY** |
| Chr9_148 | cOR1P2/OR3A2/OR01E11/OR08H10 | Olfactory receptors |
| Chr20_212 | ATXN7 | Retinal degeneration |
| Chr25_268 | PDE6D | Retinal rod cells |
| Chr27_286 | BHLHE41 | Mammalian circadian clock. Skeletal muscle differentiation. |
|  |  | **BRAIN AND COGNITION** |
| Chr23_234 | CLSTN2 | Memory |
| Chr17_202 | CTNNA2 | Excitement seeking, risk taking |
| Chr21_222 | TPH1 | Serotonin biosynthesis |
| Chr23_234 | CLSTN2 | Memory |

Table S6. SNP loci identified as outliers by BayeScan but not associated with environmental variables included in this study. Outliers without any functional genes identified in the surrounding 1 Mb regions are highlighted in bold. Pairwise comparisons for: B – Balkan-Dinaric; C – Carpathian Mountains.; U – Ukrainian Steppe; N – Northcentral Europe. 4P: across all four clusters.

| **SNP^1^** | **4P log10(PO)** | **4P FDR^2^** | **BC log10(PO)** | **BC FDR^2^** | **BU log10(PO)** | **BU FDR^2^** | **CU log10(PO)** | **CU FDR^2^** | **NB log10(PO)** | **NB FDR^2^** | **NC log10(PO)** | **NC FDR^2^** | **NU log10(PO)** | **NU FDR^2^** |
| --- | --- | --- | --- | --- | --- | --- | --- | --- | --- | --- | --- | --- | --- | --- |
| **21** | **0.907** | **0.054** |  |  |  |  |  |  |  |  |  |  |  |  |
| 54 | 1.130 | 0.031 |  |  |  |  |  |  |  |  |  |  |  |  |
| **126** | **0.640** | **0.090** |  |  |  |  |  |  |  |  |  |  |  |  |
| **130** | **0.580** | **0.097** |  |  |  |  |  |  |  |  |  |  |  |  |
| **144** | **0.908** | **0.052** |  |  |  |  |  |  |  |  |  |  |  |  |
| 145 | 0.904 | 0.058 |  |  |  |  |  |  |  |  |  |  |  |  |
| 185 | 1.059 | 0.036 |  |  |  |  |  |  |  |  |  |  |  |  |
| **193** | **0.746** | **0.079** |  |  |  |  |  |  |  |  |  |  |  |  |
| **205** | **0.712** | **0.082** |  |  |  |  |  |  |  |  |  |  |  |  |
| 221 | 0.697 | 0.085 |  |  |  |  |  |  |  |  |  |  |  |  |
| 227 | 0.749 | 0.077 |  |  |  |  |  |  |  |  |  |  |  |  |
| **240** | **0.906** | **0.055** |  |  |  |  |  |  |  |  |  |  |  |  |
| 273 | 0.704 | 0.084 |  |  |  |  |  |  |  |  |  |  |  |  |
| **320** | **0.755** | **0.076** |  |  |  |  |  |  |  |  |  |  |  |  |
| 334 | 0.861 | 0.062 |  |  |  |  |  |  |  |  |  |  |  |  |
| **337** | **0.845** | **0.065** |  |  |  |  |  |  |  |  |  |  |  |  |
| **341** | **1.385** | **0.020** |  |  |  |  |  |  |  |  |  |  |  |  |
| **23** | **0.841** | **0.067** | **1.341** | **0.028** |  |  |  |  |  |  |  |  |  |  |
| 202 | 0.607 | 0.094 | 1.004 | 0.048 |  |  |  |  |  |  |  |  |  |  |
| **253** | **0.757** | **0.075** | **1.867** | **0.013** |  |  |  |  |  |  |  |  |  |  |
| 279 | 1.257 | 0.023 | 1.894 | 0.013 |  |  |  |  |  |  |  |  |  |  |
| 292 | 3.699 | 0.000 | 1.563 | 0.018 | 1.580 | 0.010 |  |  |  |  |  |  |  |  |
| **36** | **1.580** | **0.010** |  |  | **1.378** | **0.020** |  |  |  |  |  |  |  |  |
| 143 | 0.904 | 0.058 |  |  | 1.134 | 0.033 |  |  |  |  |  |  |  |  |
| 203 | 1.823 | 0.007 |  |  | 2.744 | 0.002 |  |  |  |  |  |  |  |  |
| 215 | 2.467 | 0.002 |  |  | 1.094 | 0.045 |  |  |  |  |  |  |  |  |
| **270** | **0.615** | **0.093** |  |  | **0.693** | **0.075** |  |  |  |  |  |  |  |  |
| **304** | **0.954** | **0.048** |  |  | **1.510** | **0.015** |  |  |  |  |  |  |  |  |
| 318 | 1.418 | 0.019 |  |  | 0.673 | 0.092 |  |  |  |  |  |  |  |  |
| **120** | **2.032** | **0.005** |  |  |  |  | **0.992** | **0.053** |  |  |  |  |  |  |
| **169** | **1.207** | **0.028** |  |  |  |  | **1.214** | **0.042** |  |  |  |  |  |  |
| 291 | 0.558 | 0.102 |  |  |  |  | 0.510 | 0.106 |  |  |  |  |  |  |
| **141** | **1.534** | **0.013** |  |  |  |  |  |  | **2.317** | **0.003** |  |  |  |  |
| **301** | **0.989** | **0.044** |  |  |  |  |  |  | **1.219** | **0.023** |  |  |  |  |
| **43** | **2.152** | **0.003** | **0.947** | **0.054** |  |  |  |  | **0.880** | **0.040** |  |  |  |  |
| 217 | 0.591 | 0.096 |  |  | 0.672 | 0.096 |  |  | 1.475 | 0.014 |  |  |  |  |
| **178** | **1.459** | **0.017** |  |  |  |  | **1.481** | **0.032** |  |  | **0.638** | **0.121** |  |  |
| 8 | 0.999 | 0.042 |  |  |  |  |  |  |  |  |  |  | 1.041 | 0.044 |
| 35 | 0.954 | 0.048 |  |  |  |  |  |  |  |  |  |  | 1.217 | 0.031 |
| **137** | **1.097** | **0.033** |  |  |  |  |  |  |  |  |  |  | **0.814** | **0.065** |
| **172** | **1.762** | **0.008** |  |  |  |  |  |  |  |  |  |  | **1.946** | **0.006** |
| **187** | **1.168** | **0.030** |  |  |  |  |  |  |  |  |  |  | **1.648** | **0.015** |
| 220 | 2.083 | 0.005 |  |  |  |  |  |  |  |  |  |  | 1.309 | 0.024 |
| **271** | **1.295** | **0.021** |  |  |  |  |  |  |  |  |  |  | **1.531** | **0.017** |
| **302** | **2.093** | **0.004** |  |  |  |  |  |  |  |  |  |  | **2.795** | **0.002** |
| **310** | **0.526** | **0.109** |  |  |  |  |  |  |  |  |  |  | **1.131** | **0.038** |
| **327** | **0.838** | **0.068** |  |  |  |  |  |  |  |  |  |  | **0.804** | **0.074** |
| **339** | **0.855** | **0.064** |  |  |  |  |  |  |  |  |  |  | **0.773** | **0.094** |
| 268 |  |  | 0.771 | 0.069 |  |  |  |  |  |  |  |  |  |  |
| **285** |  |  | **1.345** | **0.024** |  |  |  |  |  |  |  |  |  |  |
| **12** |  |  |  |  | **0.692** | **0.080** |  |  |  |  |  |  |  |  |
| **129** |  |  |  |  | **0.947** | **0.058** |  |  |  |  |  |  |  |  |
| **165** |  |  |  |  | **1.069** | **0.048** |  |  |  |  |  |  |  |  |
| **224** |  |  |  |  | **0.676** | **0.088** |  |  |  |  |  |  |  |  |
| 233 |  |  |  |  | 1.063 | 0.051 |  |  |  |  |  |  |  |  |
| **289** |  |  |  |  | **1.142** | **0.028** |  |  |  |  |  |  |  |  |
| **294** |  |  |  |  | **1.117** | **0.038** |  |  |  |  |  |  |  |  |
| **305** |  |  |  |  | **0.684** | **0.084** |  |  |  |  |  |  |  |  |
| 27 |  |  |  |  |  |  | 0.952 | 0.062 |  |  |  |  |  |  |
| **124** |  |  |  |  |  |  | **0.862** | **0.075** |  |  |  |  |  |  |
| **152** |  |  |  |  |  |  | **0.557** | **0.089** |  |  |  |  |  |  |
| 153 |  |  |  |  |  |  | 0.533 | 0.098 |  |  |  |  |  |  |
| **157** |  |  |  |  |  |  | **1.131** | **0.046** |  |  |  |  |  |  |
| **175** |  |  |  |  |  |  | **1.329** | **0.038** |  |  |  |  |  |  |
| **2** |  |  |  |  |  |  |  |  | **0.617** | **0.069** |  |  |  |  |
| 113 |  |  |  |  |  |  |  |  | 0.711 | 0.054 |  |  |  |  |
| **123** |  |  |  |  |  |  |  |  | **0.643** | **0.058** |  |  |  |  |
| **150** |  |  |  |  |  |  |  |  | **0.884** | **0.037** |  |  |  |  |
| **158** |  |  |  |  |  |  |  |  | **0.623** | **0.065** |  |  |  |  |
| **167** |  |  |  |  |  |  |  |  | **0.802** | **0.051** |  |  |  |  |
| 183 |  |  |  |  |  |  |  |  | 0.511 | 0.098 |  |  |  |  |
| **204** |  |  |  |  |  |  |  |  | **1.071** | **0.030** |  |  |  |  |
| 214 |  |  |  |  |  |  |  |  | 1.522 | 0.013 |  |  |  |  |
| **295** |  |  |  |  |  |  |  |  | **2.014** | **0.007** |  |  |  |  |
| **303** |  |  |  |  |  |  |  |  | **1.448** | **0.016** |  |  |  |  |
| **307** |  |  |  |  |  |  |  |  | **1.270** | **0.022** |  |  |  |  |
| **316** |  |  |  |  |  |  |  |  | **0.601** | **0.072** |  |  |  |  |
| **24** |  |  |  |  |  |  |  |  |  |  | **0.610** | **0.129** |  |  |
| 179 |  |  |  |  |  |  |  |  |  |  | 0.556 | 0.145 |  |  |
| **196** |  |  |  |  |  |  |  |  |  |  | **0.563** | **0.138** |  |  |
| **209** |  |  |  |  |  |  |  |  |  |  | **0.833** | **0.094** |  |  |
| **342** |  |  |  |  |  |  |  |  |  |  | **0.819** | **0.106** |  |  |
| **1** |  |  |  |  |  |  |  |  |  |  |  |  | **0.802** | **0.079** |
| **17** |  |  |  |  |  |  |  |  |  |  |  |  | **0.793** | **0.087** |
| **19** |  |  |  |  |  |  |  |  |  |  |  |  | **0.778** | **0.091** |
| 31 |  |  |  |  |  |  |  |  |  |  |  |  | 0.761 | 0.097 |
| **163** |  |  |  |  |  |  |  |  |  |  |  |  | **0.802** | **0.079** |
| 177 |  |  |  |  |  |  |  |  |  |  |  |  | 0.621 | 0.118 |
| **181** |  |  |  |  |  |  |  |  |  |  |  |  | **0.686** | **0.102** |
| 189 |  |  |  |  |  |  |  |  |  |  |  |  | 1.161 | 0.036 |
| 194 |  |  |  |  |  |  |  |  |  |  |  |  | 0.515 | 0.124 |
| 207 |  |  |  |  |  |  |  |  |  |  |  |  | 0.629 | 0.113 |
| 210 |  |  |  |  |  |  |  |  |  |  |  |  | 1.206 | 0.033 |
| 225 |  |  |  |  |  |  |  |  |  |  |  |  | 0.804 | 0.074 |
| **231** |  |  |  |  |  |  |  |  |  |  |  |  | **0.628** | **0.114** |
| **232** |  |  |  |  |  |  |  |  |  |  |  |  | **0.681** | **0.104** |
| **239** |  |  |  |  |  |  |  |  |  |  |  |  | **0.795** | **0.083** |
| 243 |  |  |  |  |  |  |  |  |  |  |  |  | 0.667 | 0.107 |
| **244** |  |  |  |  |  |  |  |  |  |  |  |  | **0.750** | **0.098** |
| 272 |  |  |  |  |  |  |  |  |  |  |  |  | 0.796 | 0.081 |
| **276** |  |  |  |  |  |  |  |  |  |  |  |  | **0.623** | **0.116** |
| 278 |  |  |  |  |  |  |  |  |  |  |  |  | 1.113 | 0.041 |
| 297 |  |  |  |  |  |  |  |  |  |  |  |  | 0.546 | 0.121 |
| 313 |  |  |  |  |  |  |  |  |  |  |  |  | 0.677 | 0.105 |
| **315** |  |  |  |  |  |  |  |  |  |  |  |  | **1.686** | **0.013** |
| **328** |  |  |  |  |  |  |  |  |  |  |  |  | **0.769** | **0.095** |
| **340** |  |  |  |  |  |  |  |  |  |  |  |  | **0.610** | **0.119** |

^1^Full SNP identification given in Table S2.

^2^False discovery rate threshold (q-value).

Table S7. Pairwise F_ST_-values with 95% confidence intervals for n = 59 wolves in four population cluster, across n = 353 SNP loci reported as outliers (BayeScan) or associated with environmental variables (GWAS in PLINK), calculated in HierFstat with bootstrap resampling (n = 1,000). All were significant at p < 0.001 except where labelled otherwise (#).

| Cluster (n) | Northcentral Europe (n=32) | Ukrainian Steppe (n=11) | Dinaric-Balkan (n=9) |
| --- | --- | --- | --- |
| Ukrainian Steppe  (n=11) | **0.221**  [0.193-0.248] | - | - |
| Dinaric-Balkan  (n=9) | **0.353**  [0.317-0.387] | **0.291**^#^  [0.256-0.326] | - |
| Carpathian Mountains (n=7) | **0.236**  [0.190-0.280] | **0.259**^###^  [0.222-0.295] | **0.341**^##^  [0.295-0.388] |

# p=0.002

## p=0.052

###p=0.320
